# Supplementary material for: Access to water and sanitation among people with disabilities: results from cross-sectional surveys in Bangladesh, Cameroon, India and Malawi
Source: BMJ Open. 2018 Jun 4;8(6):e020077. doi: 10.1136/bmjopen-2017-020077 (PMC5988144; doi:10.1136/bmjopen-2017-020077)
Supplement: Supplementary data [file bmjopen-2017-020077supp001.pdf]

Supplementary File 1: Ethical Approval obtained for each study

Bangladesh-1:

- Emory University Internal Review Board (Georgia, USA)
- Icddr,b Internal Review Board (Dhaka, Bangladesh)

Bangladesh-2:

- James P Grant School of Public Health Ethical Review Committee, BRAC University (Dhaka, Bangladesh)

Cameroon:

- The London School of Hygiene and Tropical Medicine Observational/ Interventions Research Ethics Committee (London, UK)
- National Ethics Committee for Research in Human Health (CNERSH, Cameroon)
- Cameroon Baptist Convention Health Board Institutional Review Board (Cameroon)

India:

- The London School of Hygiene and Tropical Medicine Observational/ Interventions Research Ethics Committee (London, UK)
- Indian Institute of Public Health Hyderabad Institutional Ethics Committee (India)
- Government of India Health Ministry Screening Committee (India)

Malawi:

- The London School of Hygiene and Tropical Medicine Observational/ Interventions Research Ethics Committee (London, UK)
- National Commission for Science and Technology (Lilongwe, Malawi)
