# Supplementary material for: Access to water and sanitation among people with disabilities: results from cross-sectional surveys in Bangladesh, Cameroon, India and Malawi
Source: BMJ Open. 2018 Jun 4;8(6):e020077. doi: 10.1136/bmjopen-2017-020077 (PMC5988144; doi:10.1136/bmjopen-2017-020077)
Supplement: Supplementary data [file bmjopen-2017-020077supp002.pdf]

Appendix 1: Formulation of the JMP Questions and disability prevalence questions used in each data set.

Bangladesh 1	
Household head report	
Question	Responses
Does this person have a disability?	Yes<br>No
If yes, type of disability	Disability caused by Polio<br>Clubfoot<br>Effects of Leprosy<br>Brain Injury and Cerebral Palsy<br>Scoliosis (hunch back)<br>Spinal Cord Injury<br>Birth defects<br>Dwarfism and Gigantism<br>Injury, burns and amputations<br>Spinal Bifida (swelling of the spinal cord)<br>Muscular Dystrophy and Muscular Atrophy (progressive weakness of muscles and body function)<br>Juvenile Arthritis, Rheumatism and other joint pains<br>Bone infections (including tuberculosis of the spine<br>Hip problems<br>Elephantiasis.<br>Mental disability<br>Disable due to overaged<br>Disable due to blind<br>Disable due to deaf<br>Disable due to paralysis<br>Others including multiple disabilities (specify)
Does the household have any access to any latrines?	Yes

	No
If yes, type of toilet facility	**(Improved sanitation facilities)**<br>[Flush or pour flush to]:<br>Flush to pit<br>Flush to Septic tank<br>Piped sewer system/ Flush to elsewhere<br>Pit latrine with slab & water seal<br>Ventilated Improved Pit (VIP) latrine<br>Composting toilet<br><br>**(Unimproved sanitation facilities)**<br>Open pit latrine<br>Hanging toilet/latrine<br>Others: Specify
Ownership of toilet facility	Household owns toilet<br>Household Shares Ownership of Toilet<br>Public<br>Other
Person with a disability self report	
Do you use the same toilet facility as other members of your household	Yes<br>No
If no, why do you use a different toilet facility from other members of your household	It is physically impossible or difficult for me to use the same facility<br>I am not allowed to use the same facility/ other people would not like it<br>I might face physical or verbal abuse<br>I would be embarrassed<br>Other [specify]
Are you usually able to use the toilet facility without you or your clothing coming into contact with faeces	Yes<br>No
Do you collect water for drinking by yourself	Yes<br>No

Do you able collect drinking water without assistance from another person	Yes<br><br>No
Do you use the same facility / place for bathing as other members of your household	Yes<br><br>No

Bangladesh 2	
Household head report	
Question	Responses
Does (x) have difficulty seeing, even if wearing glasses?	No - no difficulty<br>Yes – some difficulty<br>Yes – a lot of difficulty<br>Cannot do at all
Does (x) have difficulty walking or climbing steps?	No - no difficulty<br>Yes – some difficulty<br>Yes – a lot of difficulty<br>Cannot do at all
Does (x) have difficulty (with self-care such as) washing all over or dressing?	No - no difficulty<br>Yes – some difficulty<br>Yes – a lot of difficulty<br>Cannot do at all
Does (x) have difficulty remembering or concentrating?	No - no difficulty<br>Yes – some difficulty<br>Yes – a lot of difficulty<br>Cannot do at all
Does (x) have difficulty hearing, even if using a hearing aid?	No - no difficulty<br>Yes – some difficulty<br>Yes – a lot of difficulty<br>Cannot do at all
Using their usual (customary) language, does (x) have difficulty communicating, for example: understanding or being understood?	No - no difficulty<br>Yes – some difficulty<br>Yes – a lot of difficulty<br>Cannot do at all
What is the main source of drinking water for members of your households?	Piped water into dwelling<br>Piped water into yard/plot<br>Public tap/standpipe<br>Tube well<br>Bore hole<br>Protected well<br>Unprotected well<br>Protected spring<br>Unprotected spring<br>Rainwater<br>Tanker truck<br>Cart with small tank<br>Surface water<br>Bottled water

	Other: specify
How many minute does it take to go to the collection point, get water, and come back? (might be 0 min if piped water into dwelling, plot or yard)	No of minutes
What kind of toilet facility do members of your household usually use?	Flush or pour flush to piped sewer /septic tank/pit latrine<br>Flush or pour flush to elsewhere/unknown<br>Ventilated improved pit latrine<br>Pit latrine with slab<br>Pit latrine without slab/open pit<br>Composting toilet<br>Bucket<br>Hanging toilet/ hanging latrine<br>No facilities or bush or field<br>Other specify
Do you share this facility with other households?	No<br>Yes, with neighbours and other people I know (Shared toilet)<br>Yes, with people I do not know (Public toilet)<br>I do not know
Person with a disability self report	
Where do you go for defecation?	Same toilet as other members go<br>Neighbours or public latrine<br>Open place<br>In the room by using a bucket/ bedpan/potty<br>Other:<br>specify________________________

Cameroon and India	
Age 2-7 – Caregiver Reported; age 8-17 self reported	
Does [name] have difficulty seeing, even when wearing his/her glasses?	No - no difficulty<br>Yes – some difficulty<br>Yes – a lot of difficulty<br>Cannot do at all
Does [name] have difficulty hearing, even when using his/her hearing aid(s)?	No - no difficulty<br>Yes – some difficulty<br>Yes – a lot of difficulty<br>Cannot do at all
Compared with children of the same age, does [name] have difficulty walking	No - no difficulty<br>Yes – some difficulty<br>Yes – a lot of difficulty<br>Cannot do at all
Age 2-4 Does [name] have difficulty understanding you?	No - no difficulty<br>Yes – some difficulty<br>Yes – a lot of difficulty

	Cannot do at all
Age 2-4 Do you have difficulty understanding what your child wants?	No - no difficulty<br>Yes – some difficulty<br>Yes – a lot of difficulty<br>Cannot do at all
Age 5+ Compared with children of the same age and using [his/her] usual language, does [name] have difficulty understanding other people	No - no difficulty<br>Yes – some difficulty<br>Yes – a lot of difficulty<br>Cannot do at all
Age 5+ Compared with children of the same age and using [his/her] usual language,<br>does [name] have difficulty being understood by other people?	No - no difficulty<br>Yes – some difficulty<br>Yes – a lot of difficulty<br>Cannot do at all
Age 5+ Compared with children of the same age, does [name] have difficulty with self-care<br>such as feeding or dressing him/herself?	No - no difficulty<br>Yes – some difficulty<br>Yes – a lot of difficulty<br>Cannot do at all
Age 2-3 Compared with children of the same age, does [name] have difficulty learning<br>the names of common objects?	No - no difficulty<br>Yes – some difficulty<br>Yes – a lot of difficulty<br>Cannot do at all
Age 3+ Compared with children of the same age, does [name] have difficulty learning to do new things?	No - no difficulty<br>Yes – some difficulty<br>Yes – a lot of difficulty<br>Cannot do at all
Age 5+ Compared with children of the same age, does [name] have difficulty remembering things that they have learned?	No - no difficulty<br>Yes – some difficulty<br>Yes – a lot of difficulty<br>Cannot do at all
Age 18+ Individual Self Report	
Question	Responses
Do you have difficulty seeing, even if wearing glasses?	No - no difficulty<br>Yes – some difficulty<br>Yes – a lot of difficulty<br>Cannot do at all
Do you have difficulty hearing, even if using a hearing aid?	No - no difficulty<br>Yes – some difficulty<br>Yes – a lot of difficulty<br>Cannot do at all
Do you have difficulty walking or climbing steps?	No - no difficulty<br>Yes – some difficulty<br>Yes – a lot of difficulty<br>Cannot do at all
Do you have difficulty remembering or concentrating?	No - no difficulty<br>Yes – some difficulty<br>Yes – a lot of difficulty

	Cannot do at all
Do you have difficulty (with self-care such as) washing al over or dressing?	No - no difficulty<br>Yes – some difficulty<br>Yes – a lot of difficulty<br>Cannot do at all
Using their usual (customary) language, does (x) have difficulty communicating, for example: understanding or being understood?	No - no difficulty<br>Yes – some difficulty<br>Yes – a lot of difficulty<br>Cannot do at all
Do you have difficulty raising a 2 liter bottle of water or soda from waist to eye level?	No - no difficulty<br>Yes – some difficulty<br>Yes – a lot of difficulty<br>Cannot do at all
Do you have difficulty using [your/his/her] hands and fingers, such as picking up small objects, for example, a button or pencil, or opening or closing containers or bottles?	No - no difficulty<br>Yes – some difficulty<br>Yes – a lot of difficulty<br>Cannot do at all
What is the main source of drinking water for members of your households?	Piped water into dwelling<br>Piped water into yard/plot<br>Public tap/standpipe<br>Tube well<br>Bore hole<br>Protected well<br>Unprotected well<br>Protected spring<br>Unprotected spring<br>Rainwater<br>Tanker truck<br>Cart with small tank<br>Surface water<br>Bottled water<br>Other: specify
Vision Screen	Presenting Visual Acuity <6/18 in better eye
Hearing Screen	Average Pure Tone Audiometry A-weighted decibel reading of >35 in better ear (children) or >40 (adults)
Musculoskeletal impairment screen	Physiotherapist observed effects on the ability of the musculoskeletal system to function as a whole, categorized as moderate or severe
Clinical Depression (18+)	Composite score of 20+ using Patient Health Questionnaire (PHQ9)
What kind of toilet facility do members of your household usually use?	Flush toilet<br>Traditional latrine<br>Ventilation improved pit latrine<br>Bowl/Bucket<br>No Toilet<br>Other: specify
Do you share this facility with other households?	Used only by your household

	Shared with other households<br>Public /Communal/ Community latrine
Do you use the same toilet facility as other members of your household	Yes<br>No
If no, why not?	It would be physically impossible<br>I'm not allowed/others would not like it<br>I might face verbal or physical abuse<br>I would be embarrassed<br>Other: specify
Are you usually able to use the toilet facility without you or your clothing coming into contact with faeces	Yes<br><br>No
What is the main source of drinking water for members of your households?	Private Pipeline<br>Private well<br>Public tap/standpipe<br>Public well<br>Neighbours<br>Water Vendor<br>Spring<br>River/stream/lake<br>Rainwater<br>Other: Specify
How long does it take to go there, get water and come back?	Number of minutes
Do you collect water for drinking?	Yes<br>No
Are you able to access drinking water at home without assistance?	Yes<br>No
Have you had diarrhea in the past four weeks?	Yes<br>No

Malawi	
Age 2-7 – Caregiver Reported; age 8-17 self reported	
Does [name] have difficulty seeing, even when wearing his/her glasses?	No - no difficulty<br>Yes – some difficulty<br>Yes – a lot of difficulty<br>Cannot do at all
Does [name] have difficulty hearing, even when using his/her hearing aid(s)?	No - no difficulty<br>Yes – some difficulty<br>Yes – a lot of difficulty<br>Cannot do at all
Compared with children of the same age, does [name] have difficulty walking	No - no difficulty<br>Yes – some difficulty<br>Yes – a lot of difficulty

	Cannot do at all
Age 2-4 Does [name] have difficulty understanding you?	No - no difficulty<br>Yes – some difficulty<br>Yes – a lot of difficulty<br>Cannot do at all
Age 2-4 Do you have difficulty understanding what your child wants?	No - no difficulty<br>Yes – some difficulty<br>Yes – a lot of difficulty<br>Cannot do at all
Age 5+ Compared with children of the same age and using [his/her] usual language, does [name] have difficulty understanding other people	No - no difficulty<br>Yes – some difficulty<br>Yes – a lot of difficulty<br>Cannot do at all
Age 5+ Compared with children of the same age and using [his/her] usual language,<br>does [name] have difficulty being understood by other people?	No - no difficulty<br>Yes – some difficulty<br>Yes – a lot of difficulty<br>Cannot do at all
Age 5+ Compared with children of the same age, does [name] have difficulty with self-care<br>such as feeding or dressing him/herself?	No - no difficulty<br>Yes – some difficulty<br>Yes – a lot of difficulty<br>Cannot do at all
Age 2-3 Compared with children of the same age, does [name] have difficulty learning<br>the names of common objects?	No - no difficulty<br>Yes – some difficulty<br>Yes – a lot of difficulty<br>Cannot do at all
Age 3+ Compared with children of the same age, does [name] have difficulty learning to do new things?	No - no difficulty<br>Yes – some difficulty<br>Yes – a lot of difficulty<br>Cannot do at all
Age 5+ Compared with children of the same age, does [name] have difficulty remembering things that they have learned?	No - no difficulty<br>Yes – some difficulty<br>Yes – a lot of difficulty<br>Cannot do at all
Age 18+ Individual Self Report if present or household head report if not	
Question	Responses
Do you have difficulty seeing, even if wearing glasses?	No - no difficulty<br>Yes – some difficulty<br>Yes – a lot of difficulty<br>Cannot do at all
Do you have difficulty hearing, even if using a hearing aid?	No - no difficulty<br>Yes – some difficulty<br>Yes – a lot of difficulty<br>Cannot do at all
Do you have difficulty walking or climbing steps?	No - no difficulty<br>Yes – some difficulty<br>Yes – a lot of difficulty

	Cannot do at all
Do you have difficulty remembering or concentrating?	No - no difficulty<br>Yes – some difficulty<br>Yes – a lot of difficulty<br>Cannot do at all
Do you have difficulty (with self-care such as) washing al over or dressing?	No - no difficulty<br>Yes – some difficulty<br>Yes – a lot of difficulty<br>Cannot do at all
Using their usual (customary) language, does (x) have difficulty communicating, for example: understanding or being understood?	No - no difficulty<br>Yes – some difficulty<br>Yes – a lot of difficulty<br>Cannot do at all
Do you have difficulty raising a 2 liter bottle of water or soda from waist to eye level?	No - no difficulty<br>Yes – some difficulty<br>Yes – a lot of difficulty<br>Cannot do at all
Do you have difficulty using [your/his/her] hands and fingers, such as picking up small objects, for example, a button or pencil, or opening or closing containers or bottles?	No - no difficulty<br>Yes – some difficulty<br>Yes – a lot of difficulty<br>Cannot do at all
Does your household have a latrine?	Yes<br>No
If yes, what type of latrine is it?	Flush to pit<br>Ventilation improved pit latrine<br>Pit latrine without slab<br>Pit latrine without slab/Open pit<br>Other: specify
Do you share this facility with other households?	Used only by your household<br>Shared with other households<br>Public /Communal/ Community latrine
Do you use the same toilet facility as other members of your household	Yes<br>No
If no, why not?	It would be physically impossible<br>I'm not allowed/others would not like it<br>I might face verbal or physical abuse<br>I would be embarrassed<br>Other: specify
Are you usually able to use the toilet facility without you or your clothing coming into contact with faeces or urine	Yes<br><br>No
What is the main source of drinking water for members of your household?	Piped into dwelling<br>Piped into compound, yard or plot<br>Piped into neighbours<br>Public tap/standpipe<br>Tubewell/borewell<br>Protected well<br>Unprotected well<br>Protected Spring

	Unprotected Spring<br>Rainwater<br>Tanker Truck<br>Cart with small tank/drum<br>Surface Water<br>Bottled Water<br>Other: Specify
How long does it take for someone to walk to the water source, collect water and come back?	Inside house<br><15 minutes<br>15 – 30 minutes<br>30 minutes – 1 hour<br>>1 hour
Do you collect water for drinking, cooking and other purposes such as for bathing, hand washing and cleaning?	Yes<br>No
If no, why not?	It would be physically impossible<br>I'm not allowed/others would not like it<br>I might face verbal or physical abuse<br>I would be embarrassed<br>Other: specify
Are you able to access drinking water at home without assistance?	Yes<br>No
Has anyone in your household had diarrhea in the past 7 days?	Yes<br>No
