# Supplementary material for: Access to water and sanitation among people with disabilities: results from cross-sectional surveys in Bangladesh, Cameroon, India and Malawi
Source: BMJ Open. 2018 Jun 4;8(6):e020077. doi: 10.1136/bmjopen-2017-020077 (PMC5988144; doi:10.1136/bmjopen-2017-020077)
Supplement: Author's manuscript [file bmjopen-2017-020077.draft_revisions.pdf]

BMJ Open

BMJ Open is committed to open peer review. As part of this commitment we make the peer review history of every article we publish publicly available.

When an article is published we post the peer reviewers' comments and the authors' responses online. We also post the versions of the paper that were used during peer review. These are the versions that the peer review comments apply to.

The versions of the paper that follow are the versions that were submitted during the peer review process. They are not the versions of record or the final published versions. They should not be cited or distributed as the published version of this manuscript.

BMJ Open is an open access journal and the full, final, typeset and author-corrected version of record of the manuscript is available on our site with no access controls, subscription charges or pay-per-view fees (http://bmjopen.bmj.com).

If you have any questions on BMJ Open's open peer review process please email info.bmjopen@bmj.com

BMJ Open

Access to water and sanitation amongst people with disabilities: results from cross-sectional surveys in Bangladesh, Cameroon, India and Malawi

Journal:	*BMJ Open*
Manuscript ID	bmjopen-2017-020077
Article Type:	Research
Date Submitted by the Author:	11-Oct-2017
Complete List of Authors:	Mactaggart, Islay; London School of Hygiene and Tropical Medicine,<br>Schmidt, Wolf-Peter; London School of Hygiene & Tropical Medicine,<br>Bostoen, Kristof; IRC<br>Chunga, Joseph; Centre for Social Research, University of Malawi,<br>Danquah, Lisa; Oxford University, Centre for Tropical Medicine and Global Health<br>Halder, Amal; icddr,b, Environmental Interventions Unit<br>Parveen Jolly, Saira; Research and Evaluation Division, BRAC<br>Polack, Sarah; London School of Hygiene & Tropical Medicine, Dept of Clinical Research<br>Rahman , Mahfuzar ; Research and Evaluation Division, BRAC,<br>Snel, Marielle; World Vision International<br>Kuper, Hannah; The London School of Hygiene & Tropical Medicine, Clinical Research<br>Biran, Adam; London School of Hygiene and Tropical Medicine, International Centre for Evidence in Disability
Keywords:	disability, population-based surveys, water, sanitation and hygiene

SCHOLARONE™
Manuscripts

Access to water and sanitation amongst people with disabilities: results from cross-sectional surveys in Bangladesh, Cameroon, India and Malawi

Authors: Islay Mactaggart[1], Wolf-Peter Schmidt[2], Kristof Bostoen[3], Joseph Chunga[4], Lisa Danquah[5], Amal Krishna Halder[6], Saira Parveen Jolly[7], Sarah Polack[1], Mahfuzar Rahman[7], Marielle Snel[8], Hannah Kuper[1], Adam Biran[2]

Corresponding author:

Ms. Islay Mactaggart

LSHTM

Keppel Street, London, WC1E 7HT, UK

E: Islay.mactaggart@lshtm.ac.uk

T: +44 (0) 2079588334

Affiliations:

[1]International Centre for Evidence in Disability, London School of Hygiene & Tropical Medicine, London UK

[2]Environmental Health Group, London School of Hygiene & Tropical Medicine, London UK

[3]Monitoring[4] chΔnge and IRC-Associate, the Hague Netherlands

[4]Centre for Social Research, University of Malawi, Zombo Malawi

[5] Centre for Tropical Medicine and Global Health, Oxford University, UK

[6]icddr,b, Dhaka Bangladesh

[7]Resarch and Evaluation Division, BRAC, Dhaka Bangladesh

[8] World Vision International, Geneva Switzerland

For peer review only - http://bmjopen.bmj.com/site/about/guidelines.xhtml

ABSTRACT

Objectives: To assess access to adequate Water, Sanitation and Hygiene (WASH) among people with disabilities at both the household and individual level.

Design: Cross-sectional surveys.

Setting: Data were included from five cross-sectional studies conducted at the district or regional level - two discrete studies in Bangladesh (Bangladesh-1 and Bangladesh-2), and one each in Cameroon, Malawi, and India.

Participants: A total of 99,252 individuals were sampled across the five datasets (ranging between 3567 and 75,767 per dataset), including 2494 people with disabilities (93 –1374 per dataset). Disability status was determined using iterations of the Washington Group Short Set in 4 surveys, and household head-reported disability (yes/no) in Bangladesh-1.

Outcome: Prevalence of access to water, sanitation and hygiene at household and individual level

Data analysis: Age and sex disaggregated disability prevalence estimates were calculated accounting for survey design. The UNICEF/WHO Joint Monitoring Programme (JMP) drinking water and sanitation definitions were used to classify household water and sanitation facilities as improved or unimproved. Multivariable logistic regression was undertaken to compare access between households with and without a person with a disability, and to identify predictors of access amongst persons with disabilities.

Results: There were no differences in access to improved sanitation or water sources or frequency of reported diarrhoeal episodes between households with and without members with disabilities in any of the five datasets. In Bangaldesh-2, households including a person with a disability were more likely to share their facility with other households (Odds Ratio [OR] =1.3, 95% Confidence Interval [95% CI] 1.1 – 1.5, p-value <0.001). Households with people with disabilities were on average more likely to spend more than 30 minutes (round trip) fetching household drinking water than households without a disabled member in both Cameroon (OR=1.8, 1.0 – 3.4, p-value <0.05) and India (OR=2.3, 1.2 – 4.7, p-value <0.05), although there were no differences in the remaining datasets. Within the household, people with disabilities reported experiencing difficulties in collecting water themselves (23% - 80% unable to collect water themselves) or accessing the same sanitation facilities as other household members, particularly without coming into contact with faeces (up to 47% of people with disabilities in Bangladesh-2). These difficulties were most marked for people with more severe impairments.

Conclusions: People with disabilities may not have poorer access to WASH at the household level, but may have poorer quality of access to WASH services within their households. Further programmatic work is needed to ensure WASH facilities are inclusive of people with disabilities.

For peer review only - http://bmjopen.bmj.com/site/about/guidelines.xhtml

Strengths and limitations of this study

- The surveys were relatively large and conducted in geographically different areas.
- Detailed information was collected on access to WASH and the presence of disabilities in different settings.
- Different methods and disability definitions were used across the surveys, making comparison of prevalence difficult.
- Each survey was conducted at the state or regional level and the estimates derived therefore do not constitute national estimates.
- Comparative data on indicators of the quality of access to WASH amongst people without disabilities were not collected in any of the studies (e.g. could hygiene facilities be used without incurring pain).

For peer review only

INTRODUCTION

Access to clean water, adequate sanitation and hygiene (WASH) for the prevention or mitigation of faeco-oral infections is a fundamental component of public health[1-4]. This is underlined by the focus of Sustainable Development Goal 6: Ensure access to water and sanitation for all[5]. 2015 estimates suggest that 91% of the global population now use an improved drinking water source (protected from outside contamination) and 68% use an improved sanitation facility (one that separates human excreta from human contact), with 1.5% of the current global disease burden attributed to unimproved water and sanitation [2 6-8]. However, these aggregate global figures may mask vulnerabilities within specific populations [9 10].

Persons with disabilities are described in the United Nations Convention on the Rights of Persons with Disabilities (UNCRPD) as those who "experience long-term physical, mental, intellectual or sensory impairments which, in interaction with various barriers, may hinder their full and effective participation in society on an equal basis with others" [11]. Persons with disabilities constitute up to 15% of the global population, and are asserted to experience poorer access to WASH, although few quantitative data exist [12]. These assertions are grounded in the limited, but growing, body of evidence that associates disability status with greater risk of widespread exclusion from education and economic productivity, alongside poor access to health care in multiple countries and contexts [12-14]. A review by Groce et al. (2011) lists potential barriers to WASH amongst persons with disabilities in the spheres of technical access barriers (such as facility structure and distance to facilities), social barriers related to stigma or abuse, and communication barriers [15], and these are supported by qualitative research [16]. The presence of social barriers, alongside the interrelationships between WASH and poverty, and poverty and disability, may also impact on WASH access within the household. Many censuses and household surveys have collected data on both WASH access and disability, however, few surveys have reported on the association. WASH access is usually only measured at the household level, and existing studies have used inconsistent definitions of disability. Quantitative data therefore remain lacking on whether persons with disabilities live in households that have poorer access to WASH, and whether persons with disabilities have poorer WASH access than other members of their household.

To address these gaps in knowledge, we analysed data from five cross-sectional, population-based studies conducted at the district or regional level that had collected data on disability and access to, and experience of, WASH. These studies were conducted in: Cameroon, Malawi, India, and Bangladesh (two discrete studies). This secondary analysis is opportunistic, based on data available. However, the inclusion of data from two African and two South-East Asian settings allows a focus on the regions with lowest access to improved facilities globally[17].

We explored 1) whether households including persons with disabilities have different access to WASH compared to households without disabled members; 2) whether persons with disabilities have different access to WASH compared to other members of their household; and 3) which factors predict access to WASH among persons with disabilities.

For peer review only

For peer review only - http://bmjopen.bmj.com/site/about/guidelines.xhtml

METHODS

We analysed data from five cross-sectional, population-based studies conducted at the district or regional level that had collected data on disability and access to WASH. These studies were conducted between 2013 - 2015 in: Cameroon, Malawi, India, and Bangladesh (two discrete studies).

Table 1 provides a comparative overview of the datasets in each study, including definitions of disability and WASH used.

Each of the studies comprised a single or multiple district, population-based survey. One of the studies conducted a census, that is, included all individuals living within the defined geographical area (Bangladesh-2). The remaining four studies utilized cluster sampling to select a random sample of participants. The samples were not nationally representative and are referred to by their country of origin for brevity. In all settings a household-level questionnaire was administered and additional questionnaire modules were used for people indentified through the survey as having disabilities. In Cameroon, India and Malawi a nested case-control study was undertaken, whereby cases with disabilities and age-sex-cluster-matched controls without disabilities were selected for further structured interview. In all settings written informed consent was collected before the questionnaire was administered, and ethical approval was obtained from the relevant boards. These include:

Bangladesh-1:

- Emory University Internal Review Board (Georgia, USA)
- Icddr,b Internal Review Board (Dhaka, Bangladesh)

Bangladesh-2:

- James P Grant School of Public Health Ethical Review Committee, BRAC University (Dhaka, Bangladesh)

Cameroon:

- The London School of Hygiene and Tropical Medicine Observational/ Interventions Research Ethics Committee (London, UK)
- National Ethics Committee for Research in Human Health (CNERSH, Cameroon)
- Cameroon Baptist Convention Health Board Institutional Review Board (Cameroon)

India:

- The London School of Hygiene and Tropical Medicine Observational/ Interventions Research Ethics Committee (London, UK)
- Indian Institute of Public Health Hyderabad Institutional Ethics Committee (India)
- Government of India Health Ministry Screening Committee (India)

Malawi:

- The London School of Hygiene and Tropical Medicine Observational/ Interventions Research Ethics Committee (London, UK)
- National Commission for Science and Technology (Lilongwe, Malawi)

For peer review only - http://bmjopen.bmj.com/site/about/guidelines.xhtml

Disability assessment

Four of the five surveys (Bangladesh-2, Cameroon, India and Malawi) used iterations of the Washington Group Short Set (WGSS) questions to screen for disability in the population (see Appendix 1 for full question sets). [18 19]. This questionnaire assesses disability status via reported limitation in core functional domains (such as seeing, hearing and self-care) on a 4-point scale ("no difficulty", "some difficulty", "a lot of difficulty" and "cannot do at all"). Participants reporting "a lot of difficulty" "cannot do at all" for any item in the questionnaire were considered to have a disability. In Cameroon and India, the Washington Group screen was supplemented with clinical screening to detect the presence of moderate or severe physical, visual or hearing impairments or severe health conditions (epilepsy and depression) using pre-validated screening methods and thresholds [20-23]. Conversely, Bangladesh-1 used a binary screen for disability whereby disability status was reported by the household head for each member of their household as "Disability – Yes or No". If the household head reported that a member of their household had a disability, they were asked to identify the type of disability (e.g. vision, hearing). Type of disability in Bangladesh-1 also included "age-related", defined as related to the process of ageing.

WASH assessment

The WASH question sets used in each study were adaptations of the World Health Organisation/ United Nations Children's Fund Joint Monitoring Programme (JMP) *Core Questions on Drinking Water and Sanitation for Household Surveys* and the JMP draft intra-household Question Set (see Appendix 1 for full question sets) [24 25]. Information about WASH at the household level was collected through interview with either the head of household (Bangladesh-1, Bangladesh-2, Malawi) or with people with disabilities and matched control subjects (Cameroon, India) as follows:

- Type of sanitation facility
- Ownership of facility
- Drinking water source (excluded in Bangladesh-1)
- Time to reach water point, collect water and return to household (excluded in Bangladesh-1)
- Recent episodes of diarrhoea (Malawi: in last 7 days; India/Cameroon: previous four weeks; Bangladesh-1 and Bangladesh-2 excluded).

Individual-level WASH access data were collected from all participants identified to have a disability in the survey, and via a proxy (usually their primary caregiver or the head of the household) for those who were not able to communicate independently (except Bangladesh-2).. Individual data were not available for the Bangladesh-2 study. Across the other data sets the following data were collected from individuals:

- Use of the same sanitation facility as other household members
- Reasons if a different facility is used
- Whether the facility can be accessed without contact with faeces
- Whether drinking water can be collected by the individual
- Reasons if drinking water cannot be collected by the individual

For peer review only - http://bmjopen.bmj.com/site/about/guidelines.xhtml

- Whether water can be accessed within the household without assistance
- Whether the same bathing place is used as other household members

Other data collected

In each survey the household head (Bangladesh-1, Bangladesh-2, Malawi) or participants with disabilities and matched control subjects (Cameroon, India) reported on the demographic composition of the household and provided data on socio-economic status (e.g. asset ownership, material of the roof/floor/wall, income).

For peer review only

For peer review only - http://bmjopen.bmj.com/site/about/guidelines.xhtml

Table 1: Study Methods					
	**Bangladesh-1 (icddr,b)**	**Bangladesh-2 (IRC-BRAC)**	**Cameroon (ICED, LSHTM)**	**India (ICED, LSHTM)**	**Malawi (collaboration[1])**
**Geographical Location**	177 sub-districts (upazillas) across Bangladesh (est. population size 750, 000)	Rangpur and Rajshahi Districts, Bangladesh (est. population size 34,149,858)	Fundong Health District, North-West Region (est. population size 125,604)	Mahbubnager District, Telegana State (est. population size 4,053,028)	Rumphi District, Northern Region (est. population size 203,054)
**Year of data collection**	2014	2015	2013	2014	2014
**Sample size (response rate %)**	1,248 households (90%)	20,000 households (98%)	4,080 individuals (87%)	4,080 individuals (88%)	1,800 households (100%)
**Sampling strategy**	Two-stage Cluster Randomised Sampling with Probability Proportionate to Size, stratified by household wealth category	Population census	Two-stage Cluster Randomised Sampling with Probability Proportionate to Size, stratified by urban and rural	Two-stage Cluster Randomised Sampling with Probability Proportionate to Size, stratified by urban and rural	Two-stage Cluster Randomised Sampling across 4 purposively selected administration units[2]
**Study definition of disability Status**	Yes/No reported by household head followed by list of conditions	Washington Group short set reported by individual if present, or female household head if not	Washington Group short set+2 criteria (Self report unless <8) or clinical impairment	Washington Group short set+2 (Self report unless <8) or clinical impairment	Washington Group extended set (report by household head or primary caregiver)
**Population-level WASH Data**	*Yes – sanitation only*	*Yes*	*No –data for case and control households only*	*No –data for case and control households only*	*Yes*
**Household-level WASH data**	*Yes – sanitation only*	*Yes*	*Yes – data for case and control households only*	*Yes – data for case and control households only*	*Yes*
**Individual level WASH data**	*Yes – people with disabilities only*	*Yes – sanitation only*	*Yes – people with disabilities only*	*Yes – people with disabilities only*	*Yes – people with disabilities only*
[1] Environmental Health Group (EHG) at the London School of Hygiene & Tropical Medicine (LSHTM), the Water Engineering and Development Centre (WEDC) at Loughborough					

For peer review only

For peer review only - http://bmjopen.bmj.com/site/about/guidelines.xhtml

University, Mzuzu University and theCentre for Social Research at the University of Malawi; [2]Purposive selection criteria: limited exposure to sanitation promotion and relative ease of access

For peer review only

For peer review only - http://bmjopen.bmj.com/site/about/guidelines.xhtml

Data Analysis

Data were analysed in Stata 12.0[26]. Descriptive statistics were used to describe each dataset.

Disability prevalence estimates for each study area were determined using the svy command to account for clustering in each of the survey designs. Severity of disability was calculated for all datasets except Bangladesh-1 (which did not include a measure of severity) as follows:

- Moderate: those with moderate clinical impairments or reporting maximum "a lot of difficulty" in at least one domain of the WGSS.
- Severe: those with severe clinical impairments or reporting "cannot do" in at least one domain of the WGSS.

Socio-economic status (SES) indices were created for Bangladesh-1, Cameroon and India using principal component analysis of assets, and divided into quartiles[27]. Bangladesh-2 data were pre-coded by interviewers at the data collection stage as ultra-poor, poor and non-poor based on land ownership and livelihood category[28]. In Malawi, annual average household income was collected as a continuous variable and divided into quartiles.

Using the JMP Millennium Development definitions, household water and sanitation facilities were classified as improved or unimproved as follows:[7]

- Improved sanitation: connected to public or private sewer; connected to septic system; pour-flush latrine; simple pit latrine; ventilated improved pit latrine
- Unimproved sanitation: public or shared latrine; open pit latrine; bucket latrine
- Improved water source: piped into household; public standpipe; borehole; protected dug well; protected spring; rainwater collection
- Unimproved water source: unprotected well, unprotected spring; rivers or ponds; vendor-provided water; bottled water; tanker truck water

We assessed whether household-level access to WASH was different in households with a disabled member compared to households that included no disabled member using multivariable logistic regression. In Malawi and Bangladesh-2 these data were available for all households. In Cameroon and India, these data were available for all households that included a person with a disability, and for age-sex-cluster matched control households that did not include a person with a disability. Household-level water access data were not available for the Bangladesh-1 study.

We evaluated whether individuals with disabilities have poorer WASH access than other members of their household by describing the reported difficulties of people with disabilities in accessing the same WASH facilities as other household members (data not available for Bangladesh-2 study). Furthermore, predictors (e.g. type of impairment, age or gender) of good intra-household access to WASH among people with disabilities were explored using multivariable logistic regression for the Cameroon, India and Malawi studies.

Missing data were excluded from analyses and listed as such in relevant tables.

For peer review only

RESULTS

A total of 99,254 individuals in 24,189 households were sampled across the five datasets (ranging between 3567 and 75,767 per dataset). The age and gender distribution of the datasets are given in Table 2. In all, the population was relatively evenly distributed between males and females, and a large proportion of the sample were children or young adults. The household size ranged from an average of 3.9 in Bangladesh-2 to 7.8 in Cameroon.

Table 2: Study sample characteristics

	BGD-1	BGD-2	Cameroon	India	Malawi
**Sample size - Households**	1207	19, 627	677	875	1803
**Sample size – individuals**	7511	75767	3467	3573	8834
**Gender**					
Female	3867 (51.5%)	37,076 (48.9%)	2112 (59.2%)	1866 (52.2%)	4500 (50.9%)
**Age**[a]					
0-17	2802 (37.3%)	26,392 (34.8%)	1950 (54.7%)	1223 (34.2%)	4562 (51.6%)
18-24	909 (12.1%)	49,375 (65.2%)	317 (8.9%)	473 (13.2%)	1179 (13.3%)
25-44	2090 (27.8%)	-	572 (16.0%)	986 (27.6%)	1862 (21.1%)
45-64	1204 (16.0%)	-	396 (11.1%)	689 (19.3%)	826 (9.4%)
65+	506 (6.7%)	-	332 (9.3%)	202 (5.7%)	405 (4.6%)
**Mean household size**	6.2 (SD 3.4)	3.9 (SD 1.5)	7.8 (SD 4.8)	5.2 (SD 2.8)	4.9 (SD 2.1)

[a]Age categories restricted to 0-1, 2-9, 10-19 and 20+ in Bangladesh-2 raw data, therefore Bangladesh-2 age data refer to 0-19 and 20+

Socio-economic status did not differ between households with and without a person with a disability in Bangladesh-1, Bangladesh-2, India and Malawi (data not shown). In Cameroon, mean asset scores were significantly lower for households with people with disabilities (-0.39, SD 2.4) than amongst households without people with disabilities (0.37, 2.6).

There was a broad range in the prevalence of disability across the settings: 1.3% (95% CI 1.0 - 1.7) in Bangladesh-1; 1.9% (1.6 – 2.1) in Bangladesh-2; 2.6% (2.4 – 2.8) in Malawi; 11.0% (9.4 – 12.7) in Cameroon and 12.6% (10.9 – 14.5) in India (Table 3). Four studies used iterations of the Washington Group Questions, supplemented by clinical tools in Cameroon and India. Disability prevalence restricted to the Washington Group Short Set only was similar across settings: 1.9% (1.6 – 2.1) in Bangladesh-2; 2.6% (2.4 – 2.8) in Malawi; 11.0% (9.4 – 12.7); 5.8% (4.6 – 7.4) in Cameroon and 7.1% (5.7 – 8.9) in India.

In each study, prevalence was similar between men and women, and increased strongly with age. The most frequent type of functional limitations reported was physical (walking or climbing stairs) in each of the five datasets. The proportions of people with disabilities reporting other functional difficulties (hearing, visual or intellectual) varied across the surveys,

For peer review only - http://bmjopen.bmj.com/site/about/guidelines.xhtml

but were overall broadly similar in four of the five datasets. Bangladesh-1 included “age-related” as a type of disability, which comprised 21.5% of the distribution.

The proportion of households including one or more persons with disabilities varied substantially between 6.5% in Bangladesh-2 and 39.3% in Cameroon.

In Bangladesh-1 and Bangladesh-2, WASH access was compared between all households either with or without a person with a disability (as identified in the population-based survey). In Cameroon, India and Malawi, comparison was made between households with a person with a disability and households with age, gender and cluster matched controls only. Table 4 provides the sample characteristics for these nested case-control studies. The nested case-control studies are therefore smaller in sample size than the two Bangladesh comparative survey samples.

Table 5 presents access to WASH amongst households with and without persons with disabilities in each dataset. No significant differences in access to an improved sanitation facility were observed between households with and without a person with a disability in any of the five datasets. In Bangladesh-2, households with a person with a disability were more likely more likely to share their sanitation facility with another household than were households having no member with a disability (Odds Ratio=1.3, 95% Confidence Interval 1.1 – 1.5) but there were no other differences in household sanitation facility ownership across datasets.

There was no difference in prevalence of access to an improved water source between households with and those without a member with a disability in any of the datasets. In Bangladesh-2 and Malawi, there was also no observed relationship between the length of time taken to collect water and disability presence in the household. However, households with a person with a disability were more likely to spend more than 30 minutes fetching household water than households without a disabled member in both Cameroon (OR=1.9, 1.1 – 3.5) and India (OR=2.4, 1.2 – 4.8). No differences in frequency of reported diarrhoeal episodes were observed by individual disability status in Cameroon or India (Cameroon OR=1.2, 0.8 – 1.9; India 0.9, 0.6 – 1.4), or household disability status in Malawi (0.7, 0.4 – 1.2).

For peer review only - http://bmjopen.bmj.com/site/about/guidelines.xhtml

Table 3 – Prevalence of disability in the 5 datasets (%, 95% CI)[a]

	BGD-1	BGD-2	Cameroon	India	Malawi
**Sample size (individuals)**	**7511**	**75767**	**3467**	**3573**	**8834**
**Overall prevalence of disability[a]**	1.3 (1.0 – 1.7)	1.9 (1.6 – 2.1)	11.0 (9.4 – 12.7)	12.6 (10.9 – 14.5)	2.6 (2.4 – 2.8)
**WGSS prevalence of disability**	-	1.9 (1.6 – 2.1)	5.8 (4.6 – 7.4)	7.1 (5.7 – 8.9)	2.6 (2.4 – 2.8)
**Gender**					
- Male	1.3 (1.0 – 1.8)	2.0 (1.7 – 2.3)	10.6 (8.9 – 12.5)	12.0 (9.9 – 14.4)	2.5 (2.1 – 3.1)
- Female	1.3 (0.9 – 1.9)	1.8 (1.5 – 2.0)	11.2 (9.3 – 13.5)	13.2 (11.3 – 15.3)	2.7 (2.3 – 3.1)
**Age**					
- 2-17	0.5 (0.3 – 0.9)	1.1 (0.9 – 1.2)	4.9 (3.9 – 6.1)	3.8 (2.7 – 5.3)	1.2 (1.0 – 1.5)
- 18-49	0.5 (0.3 – 0.9)	2.3 (1.9 – 2.6)[c]	6.9 (5.3 – 9.1)	8.2 (6.0 – 11.0)	1.6 (1.3 – 2.1)
- 50+	4.7 (3.4 – 6.5)	-	33.7 (28.8 – 38.9)	38.3 (33.6 – 43.3)	11.5 (10.2 – 12.9)
**Type of functional difficulty[b]**					
- Hearing	9.7 (4.5 – 17.6)	21.1 (19.0 – 23.4)	38.3 (33.2 – 43.5)	39.3 (34.6 – 44.0)	29.0 (23.1 – 35.6)
- Visual	6.5 (2.4 – 13.5)	26.5 (24.2 -28.9)	31.4 (26.6 – 36.5)	37.8 (33.2 – 42.5)	39.2 (32.6 – 40.0)
- Physical	41.9 (31.8 – 52.6)	57.6 (55.0 – 60.3)	53.3 (48.0 – 58.5)	51.7 (46.9 – 56.5)	50.2 (43.4 – 57.1)
- Intellectual	20.4 (12.8 - 30.1)	27.4 (25.0 – 29.8)	20.2 (16.2 – 24.7)	36.8 (21.2 – 59.0)	25.3 (19.7 – 31.7)
- Age-Related	21.5 (13.7 – 31.2)	-	-	-	-
**Proportion households with at least 1 person with a disability**	7.1(5.6 – 9.0)	6.5 (5.7 – 7.5)	39.3 (34.0 – 44.7)	38.5 (34.1 – 43.1)	10.9 (9.5 – 12.4)

[a] Disability prevalence data restricted to population aged 2+ years only

[b] Functional difficulty reported amongst people classified as having a disability. These figures are not prevalence estimates and the categories are not mutually exclusive.

[c] Age categories restricted to 0-1, 2-9, 10-19 and 20+ years in Bangladesh-2 raw data therefore Bangladesh-2 age data refer to 0-19 and 20+

For peer review only - http://bmjopen.bmj.com/site/about/guidelines.xhtml

Table 4: Nested Case-Control Study Sample Characteristics in Cameroon, India and Malawi						
	**Cameroon**		**India**		**Malawi**	
	**No Disability**	**Disability**	**No Disability**	**Disability**	**No Disability[a]**	**Disability**
**Sample size (individuals)**	274	429	337	508	200	215
	**N (%)**	**N (%)**	**N (%)**	**N (%)**	**N (%)**	**N (%)**
**Age group**						
- 5-17	90 (33%)	114 (27%)	49 (15%)	67 (13%)	-	47 (22%)
- 18-49	87 (32%)	87 (20%)	160 (48%)	177 (35%)	142 (71%)	54 (25%)
- 50+	97 (35%)	228 (53%)	128 (38%)	264 (52%)	58 (29%)	113 (53%)
**Sex**						
- Male	113 (41%)	179 (42%)	163 (48%)	236 (46%)	158 (79%)	101 (47%)
- Female	161 (59%)	250 (58%)	174 (52%)	272 (54%)	42 (21%)	113 (53%)
**SES Quartile**						
- 1st Quartile (poorest)	64 (23%)	104 (24%)	67 (20%)	142 (28%)	29 (15%)	51 (24%)
- 2nd Quartile	56 (21%)	117 (27%)	87 (26%)	120 (24%)	81 (41%)	79 (37%)
- 3rd Quartile	61 (22%)	105 (24%)	94 (28%)	124 (24%)	43 (22%)	37 (17%)
- 4th Quartile (richest)	92 (34%)	103 (24%)	89 (26%)	121 (24%)	47 (24%)	46 (21%)
**Disability Type[a]**						
- Hearing	-	142 (33%)	-	189 (37%)	-	61 (29%)
- Visual	-	123 (29%)	-	176 (35%)	-	81 (48%)
- Physical	-	257 (58%)	-	296 (58%)	-	101 (47%)
- Intellectual	-	119 (28%)	-	38 (7%)	-	51 (24%)
[a]Household Head details						

For peer review only - http://bmjopen.bmj.com/site/about/guidelines.xhtml

Access to WASH among people with disabilities in comparison to their household members without disabilities is shown in Table 6. The majority of people with disabilities reported that they were able to access the same sanitation facility as other members of their household (including those households without access to any facility). Amongst those who could not, physical access issues were most commonly reported. A substantial proportion of people with disabilities were not able to access sanitation facilities without either themselves or their clothing coming into contact with faeces. Few people with disabilities in Bangladesh-1 were able to collect water from the source themselves (21%), though the sample size was relatively small (n=78), while this proportion was higher in the other surveys (52% in Malawi to 77% in India). Physical barriers and disapproval from others were the most commonly reported reasons for persons with disabilities not collecting water themselves across the datasets. Most people with disabilities reported that they could access drinking water in the house without assistance and could use the same bathing place as other household members.

Table 7 presents the correlates of intra-household WASH access amongst people with disabilities for the three datasets that included these data (Cameroon, India and Malawi). People with more severe disabilities generally had poorer WASH access (for example a much lower likelihood [OR=0.1, 95% CI 0.1 – 0.3] of using the same sanitation facility as other household members, or using the facility without coming into contact with faeces [0.2, 0.1 – 0.3]) than people with less severe disabilities. Furthermore, older people with disabilities were more likely than children with disabilities to have access to the same sanitation facilities as other household members in all three settings. There was evidence that among people with disabilities, women were more likely than men to use the same sanitation facilities as other members of the household in Malawi, and to collect water for themselves in Cameroon and Malawi.

For peer review only - http://bmjopen.bmj.com/site/about/guidelines.xhtml

Table 5: Access to WASH amongst households with and without people with disabilities

	BGD-1			BGD-2			Cameroon			India			Malawi		
	No disability n=1121	*Disability n=86*	OR (95% CI)	*No disability n=18364*	*Disability n=1284*	OR (95% CI)	*No disability n=274*	*Disability n=429*	OR (95% CI)	*No disability n=337*	*Disability n=508*	OR (95% CI)	*No disability n=200*	*Disability n=215*	OR (95% CI)
**Sanitation facility**															
Unimproved	34.5%	31.4%	Ref.	17.4%	20.3%	Ref.	92.4%	91.4%	Ref.	23.4%	23.3%	Ref.	99.5%	99.5%	Ref.
Improved	65.5%	68.6%	1.1 (0.7 – 1.8)	82.7%	79.8%	0.8 (0.7 – 1.0)	7.6%	8.6%	0.9 (0.5 – 1.6)	76.6%	76.7%	1.0 (0.7 – 1.4)	0.5%	0.5%	1.1 (0.1- 17.3)
**Ownership**															
Used only by hh	82.2%	81.4%	Ref.	68.9%	63.0%	Ref.	86.0%	87%	Ref.	52.9%	53.3%	Ref.	-	-	
Shared ownership w/ other households	8.7%	10.5%	1.2 (0.6 – 2.5)	30.2%	35.8%	1.3 (1.1 – 1.5)*	14.0%	13.0%	0.9 (0.6 – 1.4)	14.4%	13.5%	0.9 (0.6 – 1.4)	-	-	-
Public	9.1%	8.1%	0.9 (0.4 – 2.0)	0.1%	0.1%	2.6 (0.3 – 21.6)	0	0.2%	-	32.7%	33.2%	1.0 (0.7 – 1.4)	-	-	
Don't Know	-	-	-	0.9%	1.2%	1.4 (0.8 – 2.4)	0	0	-	0	0	-	-	-	
**Time to water source**[a]															
≤ 30 m.	-	-	-	0%	0%	-	94.2%	89.6%	Ref.	96.6%	92.1%	Ref.	86.5%	80.9%	Ref.
> 30 m.				100%	100%		5.8%	10.4%	1.9 (1.1 – 3.5)*	3.4%	7.9%	2.4 (1.2 – 4.8)*	13.5%	19.1%	1.5 (0.9 – 2.6)
**Water source**															
Unimproved	-	-	-	0%	0%	-	39.2%	37.3%	Ref.	23.4%	23.3%	Ref.	12.5%	11.6%	Ref.
Improved				100%	100%		60.8%	62.7%	1.1 (0.8 – 1.5)	76.6%	76.7%	1.0 (0.7 – 1.4)	87.5%	88.4%	1.1 (0.6 – 2.0)
**At least one episode of diarrhoea in the last four weeks**[b]	-	-	-	-	-	-	11.7%	13.8%	1.2 (0.8 – 1.9)	12.8%	11.8%	0.9 (0.6 – 1.4)	21.5%	17.2%	0.8 (0.5 – 1.3)

*denotes statistical significant at the 95% confidence level

[a]Round trip travel time

[b]Refers to any episode of diarrhoea in the household on a seven day recall-period in Malawi; and any episode of diarrhoea experienced by the individual respondent on a four week recall-period in India and Cameroon

For peer review only

For peer review only - http://bmjopen.bmj.com/site/about/guidelines.xhtml

Table 6 – Access to WASH amongst individuals with disabilities							
	**BGD-1**	**BGD-2**	**Cameroon**	**India**	**India – Open Def. only**	**Malawi**	**Malawi – Open Def. only**
	*n=78**	*n=1374*	*n=429*	*n=508*	*n=274*	*n=215*	*n=36*
**Use same sanitation facility as other household members**	81%	82.0%	90%	95%	94%	94.4%	89%
**Reasons for using a different facility[a]**							
Physically impossible	93%		73%	28%	19%	33%	43%
Others would not like it/not allowed			5%	0	0	17%	0
Physical or verbal abuse		-	0	0	0	17%	0
I would be embarrassed			0	0	0	8%	0
Other	7%		23%	72%	81%	25%	57%
**Can access sanitation facility without contact with faeces**	53%	-	86%	58%	55%	86%	83%
**Can collect drinking water themselves**	21%	-	69%	77%	-	53%	67%
**Reasons cannot collect drinking water themselves**							
Physically impossible	50%		80%	86%		74%	
Others would not like it/not allowed	50%		13%	5%	-	8%	-
Physical or verbal abuse	0		0	0		3%	
I would be embarrassed	0		0	0		5%	
Other	0		8%	9%		10%	
**Can access drinking water at home without assistance**	88%[a]	-	86.%[a]	59%[a]	-	84%	81%
**Use same Bathing Source as other household members**	81%[a]	-	98%[a]	97%[a]	-	95%	86%

*8 households with a confirmed member with a disability excluded due to missing data

[a]This is of amongst the proportion who state they use a different facility

[b]This is of amongst the proportion who state they cannot collect water themselves

[c] This is amongst the proportion that collect water themselves

For peer review only

For peer review only - http://bmjopen.bmj.com/site/about/guidelines.xhtml

Table 7 – Age and sex adjusted correlates of WASH access amongst people with disabilities										
		Same sanitation facility as other household members			Access without contact with faeces			Collect water for themselves		
		No n (%)	Yes n (%)	Age and Sex Adj OR (95% CI)	No n (%)	Yes n (%)	Age and Sex Adj OR (95% CI)	No n (%)	Yes n (%)	Age and Sex Adj OR (95% CI)
Cameroon (n=412)	Age group									
	5 – 17	18 (45.0)	93 (25.0)	Ref.	24 (40.7)	87 (24.7)	Ref.	26 (20.2)	85 (30.0)	Ref.
	18-49	5 (12.5)	81 (21.8)	2.8 (1.0 –8.0)	11 (18.6)	75 (21.3)	1.8 (0.8 – 4.0)	19 (14.7)	67 (23.7)	1.0 (0.5 – 1.9)
	50+	17 (42.5)	198 (53.2)	2.1 (1.0 – 4.3)	24 (40.7)	191 (54.1)	2.1 (1.2 – 4.0)*	84 (65.1)	131 (46.3)	0.4 (0.3 – 0.7)*
	Gender									
	Male	24 (60.0)	150 (40.3)	Ref.	29 (49.2)	145 (41.1)	Ref.	70 (54.3)	104 (36.8)	Ref.
	Female	16 (40.0)	222 (59.7)	2.0 (1.0 – 4.0)	30 (50.9)	208 (58.9)	1.3 (0.7 – 2.3)	59 (45.7)	179 (63.3)	2.1 (1.4 – 3.3)*
	Type									
	Multiple	23 (57.5)	126 (33.9)	0.6 (0.3 – 1.3)	29 (49.2)	120 (34.0)	0.8 (0.4 – 1.5)	62 (48.1)	87 (30.7)	0.6 (0.4 – 1.1)
	Hearing	2 (5.0)	49 (13.2)	2.3 (0.5 – 10.9)	5 (8.5)	46 (13.0)	1.5 (0.5 – 4.3)	13 (10.1)	38 (13.4)	1.6 (0.8 – 3.5)
	Seeing	2 (5.0)	49 (13.2)	2.9 (0.6 – 13.8)	3 (5.1)	48 (13.6)	3.2 (0.9 – 11.3)	12 (9.3)	39 (13.8)	1.8 (0.8 – 4.0)
	Intellectual	0 (0.0)	27 (7.3)	-	0 (0.0)	27 (7.7)	-	2 (1.6)	25 (8.8)	4.5 (1.0 – 20.7)
	Physical	13 (32.5)	121 (32.5)	Ref.	22 (37.3)	112 (31.7)	Ref.	40 (31.0)	94 (33.2)	Ref.
	Severity									
	Moderate	19 (47.5)	324 (87.1)	Ref.	32 (54.2)	311 (88.1)	Ref.	89 (69.0)	254 (89.8)	Ref.
	Severe	21 (52.5)	48 (12.9)	0.1 (0.1 – 0.3)*	27 (45.8)	42 (11.9)	0.2 (0.1 – 0.3)*	40 (31.0)	29 (10.3)	0.2 (0.1 – 0.3)*
India (n=508)	Age group									
	5 – 17	9 (36.0)	58 (12.0)	Ref.	28 (13.0)	39 (13.3)	Ref.	18 (15.4)	49 (12.5)	Ref.
	18-49	11 (44.0)	166 (34.4)	2.4 (0.9 – 6.0)	75 (34.9)	102 (34.8)	1.0 (0.6 – 1.7)	27 (23.1)	150 (38.4)	2.0 (1.0 – 4.0)
	50+	5 (20.0)	259 (53.6)	8.2 (2.6 – 25.4)*	112 (52.1)	152 (51.9)	1.0 (0.6 – 1.7)	72 (61.5)	192 (49.1)	1.0 (0.5 – 1.8)
	Gender									
	Male	10 (40.0)	231 (47.8)	Ref.	98 (45.6)	143 (48.8)	Ref.	58 (49.6)	183 (46.8)	Ref.
	Female	15 (60.0)	252 (52.2)	0.7 (0.3 – 1.6)	117 (54.4)	150 (51.2)	0.9 (0.6 – 1.3)	59 (50.4)	208 (53.2)	1.1 (0.7 – 1.7)
	Type									
	Multiple	13 (52.0)	186 (38.5)	0.8 (0.3 – 2.0)	91 (42.3)	108 (36.9)	0.8 (0.5 – 1.3)	57 (48.7)	142 (36.3)	1.0 (0.6 – 1.8)
	Hearing	1 (4.0)	78 (16.2)	5.4 (0.7 – 42.7)	26 (12.1)	53 (18.1)	1.5 (0.8 – 2.5)	14 (12.0)	65 (16.6)	2.3 (1.1 – 4.6)*
	Seeing	0 (0.0)	76 (15.7)	-	35 (16.3)	41 (14.0)	0.8 (0.4 – 1.4)	9 (7.7)	67 (17.1)	3.4 (1.5 – 7.6)*
	Intellectual	1 (4.0)	16 (3.3)	0.8 (0.1 – 7.7)	7 (3.3)	10 (3.4)	2.1 (0.4 – 11.0)	4 (3.4)	13 (3.3)	0.9 (0.2 – 5.0)
	Physical	10 (40.0)	127 (26.3)	Ref.	56 (26.1)	81 (27.7)	Ref.	33 (28.2)	104 (26.6)	Ref.
	Severity									
	Moderate	17 (68.0)	364 (75.4)	Ref.	150 (69.8)	231 (78.8)	Ref.	94 (80.4)	287 (73.4)	Ref.
	Severe	8 (32.0)	119 (24.6)	0.9 (0.4 – 2.2)	65 (30.2)	62 (21.2)	0.6 (0.4 – 0.9)*	23 (19.7)	104 (26.6)	1.4 (0.8 – 2.3)

For peer review only - http://bmjopen.bmj.com/site/about/guidelines.xhtml

Malawi (n=215)	Age group									
	2 – 17	6 (50.0)	40 (19.9)	Ref.	10 (34.5)	35 (19.1)	Ref.	14 (14.0)	33 (29.0)	Ref.
	18-49	5 (41.7)	49 (24.4)	1.5 (0.4 – 5.2)	2 (6.9)	52 (28.4)	7.4 (1.5 – 36.1)*	20 (20.0)	34 (29.8)	0.7 (0.3 – 1.7)
	50+	1 (8.3)	112 (55.7)	18.2 (2.1 – 157.6)*	17 (58.6)	96 (52.5)	1.6 (0.6 – 3.8)	66 (66.0)	47 (41.2)	0.3 (0.1 – 0.6)*
	Gender									
	Male	5 (41.7)	96 (47.8)	Ref.	15 (51.7)	86 (47.0)	Ref.	52 (50.0)	49 (43.0)	Ref.
	Female	7 (58.3)	105 (52.4)	0.6 (0.2 – 2.0)	14 (48.3)	97 (53.0)	1.3 (0.6 – 2.8)	48 (48.0)	65 (57.0)	1.7 (1.0 – 3.1)
	Type									
	Multiple	6 (50.0)	67 (35.1)	Ref.	16 (55.2)	56 (32.4)	Ref.	43 (44.8)	30 (27.8)	Ref.
	Hearing	2 (16.7)	25 (13.1)	1.9 (0.3 – 11.0)	1 (3.5)	26 (15.0)	9.7 (1.1 – 82.1)*	6 (6.3)	21 (19.4)	5.0 (1.7 – 14.8)*
	Seeing	0	42 (22.0)	-	2 (6.9)	40 (23.1)	6.4 (1.4 – 30.4)*	19 (19.8)	23 (21.3)	2.0 (0.9 – 4.6)
	Intellectual	1 (8.3)	13 (6.8)	2.6 (0.3 – 25.4)	3 (10.3)	11 (6.4)	1.5 (0.3 – 7.1)	3 (3.1)	11 (10.2)	4.2 (1.0 – 17.4)
	Physical	3 (25.0)	44 (23.0)	1.1 (0.2 – 5.1)	7 (24.1)	40 (23.1)	1.5 (0.6 – 4.2)	25 (26.0)	23 (21.3)	1.5 (0.7 – 3.2)
	Severity									
	Moderate	7 (58.3)	152 (79.6)	Ref.	14 (48.3)	145 (83.8)	Ref.	62 (64.6)	97 (89.8)	Ref.
	Severe	5 (41.7)	39 (20.4)	0.4 (0.1 – 1.4)	15 (51.7)	28 (16.2)	0.2 (0.1 -0.4)*	34 (35.4)	11 (10.2)	0.2 (0.1 – 0.3)*

*denotes statistical significant at the 95% confidence level

For peer review only

For peer review only - http://bmjopen.bmj.com/site/about/guidelines.xhtml

DISCUSSION

Poorer access to WASH amongst people with disabilities across low and middle income countries is "widely acknowledged but little studied" [15]. This analysis of five cross-sectional, single or multi-district, population-based surveys provides some of the first comparable quantitative data on the relationship between disability and WASH at the individual and household level, including multi-country evidence from two African and two South-Asian settings.

No differences in access to improved latrines were observed between households with and without a person with a disability in any of the five datasets. Households with a person with a disability were more likely to have to spend over thirty minutes (round-trip) fetching water than those without at the Cameroon and Indian sites, and households including a person with a disability were more likely to use a shared sanitation facility in Bangladesh-2. The former finding may suggest that in these two settings households with one or more people with disabilities live further away from centrally located water points (i.e. community peripheries). Secondly, given that the questions were posed directly to people with disabilities in the house, it may reflect their increased time to complete the same journey as others. Shared sanitation facilities of any description were previously considered unimproved by the JMP, due to concerns of privacy and hygiene[29]. However, the 2017 update acknowledges improved shared facilities, and a recent study by Nelson et al. (2014) estimated that 37% of households in Bangladesh (similar to proportions identified in Bangladesh-2 data) utilised a shared but improved facility, and that 75% of households reported satisfaction with the facility[30 31].

Within the household, most people with disabilities used the same sanitation facility as other members of their family, but also frequently reported coming into direct contact with faeces when they did so. Furthermore, the majority of people with disabilities in Bangladesh and almost half in Malawi were unable to collect drinking water from the source themselves. These analyses also showed that older people generally had better access than children, although given the different WASH practices of children and older people it is difficult to know whether or not this was related to disability.

Overall, few differences in WASH access were observed between households that included a member with a disability and those that did not. However, there is a need to better understand the quality of WASH service accessed by people with disabilities, particularly for those whose households do not have access to improved facilities. In India, for example, almost two thirds of people with disabilities interviewed practiced open defecation, and over half of people with disabilities were unable to do this without coming into direct contact with faeces. These findings are of concern as it is well established that direct contact with faeces substantially increases risk of both diarrhoeal disease and hygiene-related stigma [32]. Several recent studies have observed survey respondents' preference for open defecation in rural India even when latrines are available, highlighting the need for clearer and sustained promotion on disability inclusive latrine use in this setting[33 34].

There was evidence that people with disabilities have more difficulties both collecting water themselves and accessing it within their homes across datasets. This has been previously linked to lower volume of water consumption, dehydration and the potential for increased morbidity [35]. These findings confirm the need for more nuanced quantitative data collection that captures the quality of access to WASH at the individual level and can inform on appropriate WASH

For peer review only - http://bmjopen.bmj.com/site/about/guidelines.xhtml

programming. This is important because previous research has suggested that WASH programmes may inadvertently exclude people with disabilities through the delivery of improved, but inaccessible facilities, highlighting the need for better quality monitoring data that records whether facilities are accessible or not [15]. Our findings show that data on WASH and disability should be collected at the individual rather than the household level in order to identify the concerns facing people with disabilities in this domain. Specifically, these findings promote the inclusion of intra-household access questions in JMP, to better understand and overcome lower quality access to WASH amongst persons with disabilities within their households.

Furthermore, additional domains may need to be included in WASH scores, focusing on items such as appropriateness and quality of WASH available with respect to people with disabilities. Ideally, surveys on disability should routinely include individual WASH data as it represents a major challenge facing people with disabilities in low and middle income countries[16].

Several meta-analyses have quantified the implications of improved WASH for exposure pathways for diarrhoeal and infectious diseases, thereby affecting disease burden in "Disability-Adjusted Life Years" (DALYS)[36 37]. However, no previous quantitative studies have explored the implications of disability for access to improved WASH, and in particular the implications for quality of WASH access. A recent qualitative study undertaken in Malawi established that needs and barriers amongst people with disabilities related to WASH varied markedly between individuals, in relation to impairment type, gender and socio-economic factors[16], with much more work needed in this area. Programme implementers and policy makers must ensure that the needs of persons with disabilities are incorporated into programme design in a comprehensive, participatory way that includes intra-household WASH access.

There were a number of limitations to the design of the analyses that need to be taken into account. Whilst response rates across datasets were high, data on the characteristics of non-responders were not available. Disability prevalence estimates varied markedly across the studies, largely because of the different methods and disability definitions used across the surveys. In Cameroon and India, a self-reported measure of disability was combined with clinical screening for specific impairments, and therefore the prevalence was higher there than in the Bangladesh-2 and Malawi studies where the self-reported measure was used in isolation. In the Bangladesh-1 study, a binary screen was applied, which may explain the low prevalence of disability in that survey. Presenting prevalence estimates based on the Washington Group Short Set (WGSS) only provided similar estimates across settings, although these were higher in the datasets where individuals self-reported (Cameroon and India) compared to those in which a household head reported on behalf of the rest of the household (Bangladesh-2 and Malawi). This finding supports the ongoing dialogue regarding the importance of comparable disability measures for use in population-based surveys, including in the measurement of childhood disability, the international recommendation for which is the use of tools by the Washington Group on Disability Statistics [38 39]. Furthermore, each survey was conducted at the state or regional level and the estimates derived therefore do not constitute national estimates. Finally, comparative data on indicators of quality access to WASH amongst controls/ people without disabilities were not collected in any of the studies. There were also important strengths. The surveys were relatively large, conducted in different geographic areas, and collected broadly consistent information on WASH.

For peer review only - http://bmjopen.bmj.com/site/about/guidelines.xhtml

CONCLUSION

No relationship was observed between household-level WASH access and the presence of a household member with a disability in five surveys conducted in low and middle income countries. However, at the individual level, whilst most people with disabilities could access the same facilities as other members of their household, this often entailed contact with faeces and required assistance from others. In particular, people who reported severe limitations experienced the least equitable intra-household WASH access. Type of limitation reported, age and gender were also predictive factors for inequitable access in certain cases. Consequently, the quality of the WASH access is likely to have been poorer among people with disabilities. This requires programmatic and research attention. Future data collection should capture quality of WASH access in a more nuanced way and collect individual level data from people with and without disabilities to make accurate comparisons. Further programmatic work is needed to improve general access to WASH, and this must be inclusive in nature to ensure that the quality of access is equitable among people with disabilities.

ACKNOWLEDGEMENTS

We gratefully acknowledge the contribution of our colleague the late James Milner. His untimely and tragic death during fieldwork in Malawi was a great loss to his friends and colleagues as well as to the scientific research community in Malawi and beyond.

FUNDING

This research has been funded in part by the Australian Aid (Australian Government Department of Foreign Affairs and Trade) through the Australian Development Research Awards Scheme under an award entitled 'Disability and its impact on safe hygiene and sanitation. The views expressed in this publication are those of the authors and not necessarily those of the Commonwealth of Australia. The Commonwealth of Australia accepts no responsibility for any loss, damage or injury resulting from reliance on any of the information or views contained in this publication.

Bangladesh-1: Funded by The Bill & Melinda Gates Foundation, Global Development Grant Number OPP1102989.

Bangladesh-2: Funded by Australian Aid (Australian Government Department of Foreign Affairs and Trade), Grant number 66469

India and Cameroon: Funded by CBM Germany, LSHTM Grant Number ITCRRH71

Malawi: Funded by Australian Aid (Australian Government Department of Foreign Affairs and Trade), Grant number 66469

COMPETING INTERESTS

There were no competing interests.

AUTHOR'S CONTRIBUTIONS

For peer review only - http://bmjopen.bmj.com/site/about/guidelines.xhtml

All authors reviewed the final draft of the manuscript and agree with the findings and conclusions.

IM was responsible for drafting the paper, conducting the data analysis, and overseeing the fieldwork in India and Cameroon.

WPS gave oversight on the data analysis and feedback on all manuscript drafts

KB contributed to the study design of the Bangladesh-2 dataset and reviewed manuscript drafts

JC oversaw data collection for the Malawi dataset

LD contributed to the study design of the Malawi dataset, oversaw data collection for the Malawi dataset and reviewed manuscript drafts

AKH was responsible for the study design of the Bangladesh-1 dataset, oversaw data collection and data management for the Bangldesh-1 dataset and reviewed manuscript drafts

SPJ contributed to the study design of the Bangladesh-2 dataset, oversaw data collection for the Bangladesh-2 dataset and reviewed manuscript drafts

SP was responsible for the study design and data collection on India and Cameroon.

MR contributed to the study design of the Bangladesh-2 dataset

MS contributed to the study design of the Bangladesh-2 dataset

HK assisted in drafting the paper, and guided the data analysis, and contributed towards designing the study in India and Cameroon.

AB was responsible for the study design of the Malawi dataset, contributed to the design of the Bangladesh-2 dataset, gave oversight on data analysis and feedback on all manuscript drafts, and coordinated access to external datasets. AB also contributed to the design of the data collection tool across all study sites.

DATA SHARING STATEMENT

Data for each dataset is available from the respective project leads on request.

REFERENCES

1. Johnston EA, Teague J, Graham JP. Challenges and opportunities associated with neglected tropical disease and water, sanitation and hygiene intersectoral integration programs. *BMC Public Health* 2015;15(1):547.
2. Prüss-Ustün A, Bartram J, Clasen T, et al. Burden of disease from inadequate water, sanitation and hygiene in low-and middle-income settings: a retrospective analysis of data from 145 countries. *Tropical Medicine & International Health* 2014;19(8):894-905.
3. Freeman MC, Ogden S, Jacobson J, et al. Integration of water, sanitation, and hygiene for the prevention and control of neglected tropical diseases: a rationale for inter-sectoral collaboration. *PLoS Negl Trop Dis* 2013;7(9):e2439.
4. Mara DD. Water, sanitation and hygiene for the health of developing nations. *Public health* 2003;117(6):452-56.
5. United Nations. Transforming our world: the 2030 Agenda for Sustainable Development. Geneva: United Nations General Assembly, 2015.

For peer review only

For peer review only - http://bmjopen.bmj.com/site/about/guidelines.xhtml

6. UNICEF and the World Health Organisation. 25 years Progress on Sanitation and Drinking Water: 2015 Update and MDG Assessment. United States of America: Available from: http://www.wssinfo.org/fileadmin/user_upload/resources/JMP-Update-report-2015_English.pdf, 2015.
7. WHO / UNICEF JMP. Refining the definitions: an ongoing process and the ladder concept Available from: http://www.wssinfo.org/definitions-methods/2015 [2015.
8. Lim SS, Vos T, Flaxman AD, et al. A comparative risk assessment of burden of disease and injury attributable to 67 risk factors and risk factor clusters in 21 regions, 1990–2010: a systematic analysis for the Global Burden of Disease Study 2010. *The lancet* 2013;380(9859):2224-60.
9. Schmidt WP. The elusive effect of water and sanitation on the global burden of disease. *Tropical Medicine & International Health* 2014;19(5):522-27.
10. Bullinger M, Schmidt S, Petersen C, et al. Assessing quality of life of children with chronic health conditions and disabilities: a European approach. *International Journal of Rehabilitation Research* 2002;25(3):197-206.
11. United Nations. Convention of the Rights of Persons with Disabilities and Optional Protocol. New York: United Nations, 2006.
12. World Health Organization. World Report on Disability Geneva: World Health Organization 2011.
13. Mitra S, Pošarac A, Vick B. Disability and poverty in developing countries: a snapshot from the world health survey: World Bank 2011.
14. Mactaggart I, Kuper H, Murthy G, et al. Assessing health and rehabilitation needs of people with disabilities in Cameroon and India. *Disability and rehabilitation* 2015:1-8.
15. Groce N, Bailey N, Lang R, et al. Water and sanitation issues for persons with disabilities in low-and middle-income countries: a literature review and discussion of implications for global health and international development. *Journal of water and health* 2011;9(4):617-27.
16. White S, Kuper H, Itimu-Phiri A, et al. A Qualitative Study of Barriers to Accessing Water, Sanitation and Hygiene for Disabled People in Malawi. *PloS one* 2016;11(5):e0155043.
17. Moe CL, Rheingans RD. Global challenges in water, sanitation and health. *Journal of water and health* 2006;4(S1):41-57.
18. Washington Group on Disability Statistics. Understanding and interpreting disability as measured using the WG short set of questions. Hyattsville, 2009.
19. Secretariat to the Convention on the Rights of Persons with Disabilities. Final Report on United Nations Expert Group Meeting on Disability Data and Statistics, Monitoring and Evaluation: The Way Forward - a Disability-Inclusive Agenda Towards 2015 and Beyond; Paris July 8-10 2014. Available from http://www.un.org/disabilities/documents/egm2014/EGM_FINAL_08102014.pdf [first accessed 04.01.15], 2014.
20. Atijosan O, Kuper H, Rischewski D, et al. Musculoskeletal impairment survey in Rwanda: design of survey tool, survey methodology, and results of the pilot study (a cross sectional survey). *BMC musculoskeletal disorders* 2007;8(1):30.
21. Lindfield R, Griffiths U, Bozzani F, et al. A rapid assessment of avoidable blindness in Southern Zambia United States: International Centre for Eye Health, London School of Hygiene and Tropical Medicine-LSHTM, London, United Kingdom. Robert.lindfield@lshtm.ac.uk; 2012 [6:[e38483]. Available from: http://ovidsp.ovid.com/ovidweb.cgi?T=JS&PAGE=reference&D=medl&NEWS=N&AN=22737211 accessed 101285081 7.
22. Smith A, Mackenzie I. WHO Ear and Hearing Disorders Survey Protocol. Geneva: World Health Organization, 1999.
23. Kroenke K, Spitzer RL. The PHQ-9: a new depression diagnostic and severity measure. *Psychiatr Ann* 2002;32(9):1-7.
24. UNICEF and the World Health Organisation. Core questions on drinking-water and sanitation for household surveys. Switzerland, available from:

For peer review only - http://bmjopen.bmj.com/site/about/guidelines.xhtml

http://www.wssinfo.org/fileadmin/user_upload/resources/1268174016-JMP_Core_Questions.pdf, 2006.
25. Meg Satterthwaite. JMP Working Group on Equity and Non-Discrimination Final Report. JMP Working Group on Equity and Non-Discrimination (END), available from: http://www.wssinfo.org/fileadmin/user_upload/resources/JMP-END-WG-Final-Report-20120821.pdf, 2012.
26. StataCorp L. Stata 12.0. *Statistics/Data Analysis* 2011;4905
27. Vyas S, Kumaranayake L. Constructing socio-economic status indices: how to use principal components analysis. *Health policy and planning* 2006;21(6):459-68.
28. BRAC. Water, Sanitation and Hygiene: Nine years of scale and innovation in Bangladesh Programme Report 2006-2015. Available from http://www.brac.net/images/WASH/BRACWASHProgrammeFinalReport20062015.pdf [accessed 18.09.17], 2016.
29. Heijnen M, Cumming O, Peletz R, et al. Shared sanitation versus individual household latrines: a systematic review of health outcomes. *PLoS One* 2014;9(4):e93300.
30. Nelson KB, Karver J, Kullman C, et al. User perceptions of shared sanitation among rural households in Indonesia and Bangladesh. *PloS one* 2014;9(8):e103886.
31. Organization WH, UNICEF. Progress on drinking water, sanitation and hygiene: 2017 update and SDG baselines. 2017
32. Murray CJ, Lopez AD. Global mortality, disability, and the contribution of risk factors: Global Burden of Disease Study. *The Lancet* 1997;349(9063):1436-42.
33. Coffey D, Gupta A, Hathi P, et al. Revealed preference for open defecation. *Economic & Political Weekly* 2014;49(38):43.
34. Gertler P, Shah M, Alzua ML, et al. How does health promotion work? Evidence from the dirty business of eliminating open defecation: National Bureau of Economic Research, 2015.
35. Manz F, Wentz A. The importance of good hydration for the prevention of chronic diseases. *Nutrition reviews* 2005;63(6):S2.
36. Prüss A, Kay D, Fewtrell L, et al. Estimating the burden of disease from water, sanitation, and hygiene at a global level. *Environmental health perspectives* 2002;110(5):537-42.
37. Gleick PH. Dirty-water: Estimated Deaths from Water-related Diseases 2000-2020: Citeseer 2002.
38. United Nations. Improvement of disability data and statistics: objectives and challenges. Conference of State Parties to the Convention on the Rights of Persons with Disabilities Eighth Session, New York 9-11 June 2015, 2015.
39. Palmer MH, D. Models and Measurement in disability: an international review. *Health Policy and Planning* 2011:1-8.

Appendix 1: Formulation of the JMP Questions and disability prevalence questions used in each data set.

Bangladesh 1	
Household head report	
Question	Responses
Does this person have a disability?	Yes<br>No
If yes, type of disability	Disability caused by Polio<br>Clubfoot

For peer review only - http://bmjopen.bmj.com/site/about/guidelines.xhtml

	Effects of Leprosy<br>Brain Injury and Cerebral Palsy<br>Scoliosis (hunch back)<br>Spinal Cord Injury<br>Birth defects<br>Dwarfism and Gigantism<br>Injury, burns and amputations<br>Spinal Bifida (swelling of the spinal cord)<br>Muscular Dystrophy and Muscular Atrophy (progressive weakness of muscles and body function)<br>Juvenile Arthritis, Rheumatism and other joint pains<br>Bone infections (including tuberculosis of the spine<br>Hip problems<br>Elephantiasis.<br>Mental disability<br>Disable due to overaged<br>Disable due to blind<br>Disable due to deaf<br>Disable due to paralysis<br>Others including multiple disabilities (specify)
Does the household have any access to any latrines?	Yes<br>No
If yes, type of toilet facility	**(Improved sanitation facilities)**<br>[Flush or pour flush to]:<br>Flush to pit<br>Flush to Septic tank<br>Piped sewer system/ Flush to elsewhere<br>Pit latrine with slab & water seal<br>Ventilated Improved Pit (VIP) latrine<br>Composting toilet<br><br>**(Unimproved sanitation facilities)**<br>Open pit latrine<br>Hanging toilet/latrine<br>Others: Specify
Ownership of toilet facility	Household owns toilet<br>Household Shares Ownership of Toilet<br>Public<br>Other
Person with a disability self report	
Do you use the same toilet facility as other members of your household	Yes<br>No
If no, why do you use a different toilet facility from other members of your household	It is physically impossible or difficult for me to use the same facility<br>I am not allowed to use the same facility/ other people would not like it<br>I might face physical or verbal abuse<br>I would be embarrassed<br>Other [specify]
Are you usually able to use the toilet facility without you or your clothing coming into	Yes<br>No

For peer review only
For peer review only - http://bmjopen.bmj.com/site/about/guidelines.xhtml

contact with faeces	
Do you collect water for drinking by yourself	Yes<br>No
Do you able collect drinking water without assistance from another person	Yes<br>No
Do you use the same facility / place for bathing as other members of your household	Yes<br>No

Bangladesh 2	
Household head report	
Question	Responses
Does (x) have difficulty seeing, even if wearing glasses?	No - no difficulty<br>Yes – some difficulty<br>Yes – a lot of difficulty<br>Cannot do at all
Does (x) have difficulty walking or climbing steps?	No - no difficulty<br>Yes – some difficulty<br>Yes – a lot of difficulty<br>Cannot do at all
Does (x) have difficulty (with self-care such as) washing all over or dressing?	No - no difficulty<br>Yes – some difficulty<br>Yes – a lot of difficulty<br>Cannot do at all
Does (x) have difficulty remembering or concentrating?	No - no difficulty<br>Yes – some difficulty<br>Yes – a lot of difficulty<br>Cannot do at all
Does (x) have difficulty hearing, even if using a hearing aid?	No - no difficulty<br>Yes – some difficulty<br>Yes – a lot of difficulty<br>Cannot do at all
Using their usual (customary) language, does (x) have difficulty communicating, for example: understanding or being understood?	No - no difficulty<br>Yes – some difficulty<br>Yes – a lot of difficulty<br>Cannot do at all
What is the main source of drinking water for members of your households?	Piped water into dwelling<br>Piped water into yard/plot<br>Public tap/standpipe<br>Tube well<br>Bore hole<br>Protected well<br>Unprotected well<br>Protected spring<br>Unprotected spring<br>Rainwater<br>Tanker truck<br>Cart with small tank<br>Surface water<br>Bottled water<br>Other: specify

For peer review only

For peer review only - http://bmjopen.bmj.com/site/about/guidelines.xhtml

How many minute does it take to go to the collection point, get water, and come back? (might be 0 min if piped water into dwelling, plot or yard)	No of minutes
What kind of toilet facility do members of your household usually use?	Flush or pour flush to piped sewer /septic tank/pit latrine<br>Flush or pour flush to elsewhere/unknown<br>Ventilated improved pit latrine<br>Pit latrine with slab<br>Pit latrine without slab/open pit<br>Composting toilet<br>Bucket<br>Hanging toilet/ hanging latrine<br>No facilities or bush or field<br>Other specify
Do you share this facility with other households?	No<br>Yes, with neighbours and other people I know (Shared toilet)<br>Yes, with people I do not know (Public toilet)<br>I do not know
Person with a disability self report	
Where do you go for defecation?	Same toilet as other members go<br>Neighbours or public latrine<br>Open place<br>In the room by using a bucket/ bedpan/potty<br>Other:<br>specify______________________

265

Cameroon and India	
Age 2-7 – Caregiver Reported; age 8-17 self reported	
Does [name] have difficulty seeing, even when wearing his/her glasses?	No - no difficulty<br>Yes – some difficulty<br>Yes – a lot of difficulty<br>Cannot do at all
Does [name] have difficulty hearing, even when using his/her hearing aid(s)?	No - no difficulty<br>Yes – some difficulty<br>Yes – a lot of difficulty<br>Cannot do at all
Compared with children of the same age, does [name] have difficulty walking	No - no difficulty<br>Yes – some difficulty<br>Yes – a lot of difficulty<br>Cannot do at all
Age 2-4 Does [name] have difficulty understanding you?	No - no difficulty<br>Yes – some difficulty<br>Yes – a lot of difficulty<br>Cannot do at all
Age 2-4 Do you have difficulty understanding what your child wants?	No - no difficulty<br>Yes – some difficulty<br>Yes – a lot of difficulty<br>Cannot do at all

For peer review only - http://bmjopen.bmj.com/site/about/guidelines.xhtml

Age 5+ Compared with children of the same age and using [his/her] usual language, does [name] have difficulty understanding other people	No - no difficulty<br>Yes – some difficulty<br>Yes – a lot of difficulty<br>Cannot do at all
Age 5+ Compared with children of the same age and using [his/her] usual language,<br>does [name] have difficulty being understood by other people?	No - no difficulty<br>Yes – some difficulty<br>Yes – a lot of difficulty<br>Cannot do at all
Age 5+ Compared with children of the same age, does [name] have difficulty with self-care<br>such as feeding or dressing him/herself?	No - no difficulty<br>Yes – some difficulty<br>Yes – a lot of difficulty<br>Cannot do at all
Age 2-3 Compared with children of the same age, does [name] have difficulty learning<br>the names of common objects?	No - no difficulty<br>Yes – some difficulty<br>Yes – a lot of difficulty<br>Cannot do at all
Age 3+ Compared with children of the same age, does [name] have difficulty learning to do new things?	No - no difficulty<br>Yes – some difficulty<br>Yes – a lot of difficulty<br>Cannot do at all
Age 5+ Compared with children of the same age, does [name] have difficulty remembering things that they have learned?	No - no difficulty<br>Yes – some difficulty<br>Yes – a lot of difficulty<br>Cannot do at all
Age 18+ Individual Self Report	
Question	Responses
Do you have difficulty seeing, even if wearing glasses?	No - no difficulty<br>Yes – some difficulty<br>Yes – a lot of difficulty<br>Cannot do at all
Do you have difficulty hearing, even if using a hearing aid?	No - no difficulty<br>Yes – some difficulty<br>Yes – a lot of difficulty<br>Cannot do at all
Do you have difficulty walking or climbing steps?	No - no difficulty<br>Yes – some difficulty<br>Yes – a lot of difficulty<br>Cannot do at all
Do you have difficulty remembering or concentrating?	No - no difficulty<br>Yes – some difficulty<br>Yes – a lot of difficulty<br>Cannot do at all
Do you have difficulty (with self-care such as) washing al over or dressing?	No - no difficulty<br>Yes – some difficulty<br>Yes – a lot of difficulty<br>Cannot do at all
Using their usual (customary) language, does (x) have difficulty communicating, for example: understanding or being understood?	No - no difficulty<br>Yes – some difficulty<br>Yes – a lot of difficulty<br>Cannot do at all
Do you have difficulty raising a 2 liter bottle of water or soda from waist to eye level?	No - no difficulty<br>Yes – some difficulty<br>Yes – a lot of difficulty

For peer review only - http://bmjopen.bmj.com/site/about/guidelines.xhtml

	Cannot do at all
Do you have difficulty using [your/his/her] hands and fingers, such as picking up small objects, for example, a button or pencil, or opening or closing containers or bottles?	No - no difficulty<br>Yes – some difficulty<br>Yes – a lot of difficulty<br>Cannot do at all
What is the main source of drinking water for members of your households?	Piped water into dwelling<br>Piped water into yard/plot<br>Public tap/standpipe<br>Tube well<br>Bore hole<br>Protected well<br>Unprotected well<br>Protected spring<br>Unprotected spring<br>Rainwater<br>Tanker truck<br>Cart with small tank<br>Surface water<br>Bottled water<br>Other: specify
Vision Screen	Presenting Visual Acuity <6/18 in better eye
Hearing Screen	Average Pure Tone Audiometry A-weighted decibel reading of >35 in better ear (children) or >40 (adults)
Musculoskeletal impairment screen	Physiotherapist observed effects on the ability of the musculoskeletal system to function as a whole, categorized as moderate or severe
Clinical Depression (18+)	Composite score of 20+ using Patient Health Questionnaire (PHQ9)
What kind of toilet facility do members of your household usually use?	Flush toilet<br>Traditional latrine<br>Ventilation improved pit latrine<br>Bowl/Bucket<br>No Toilet<br>Other: specify
Do you share this facility with other households?	Used only by your household<br>Shared with other households<br>Public /Communal/ Community latrine
Do you use the same toilet facility as other members of your household	Yes<br>No
If no, why not?	It would be physically impossible<br>I'm not allowed/others would not like it<br>I might face verbal or physical abuse<br>I would be embarrassed<br>Other: specify

For peer review only - http://bmjopen.bmj.com/site/about/guidelines.xhtml

Are you usually able to use the toilet facility without you or your clothing coming into contact with faeces	Yes<br>No
What is the main source of drinking water for members of your households?	Private Pipeline<br>Private well<br>Public tap/standpipe<br>Public well<br>Neighbours<br>Water Vendor<br>Spring<br>River/stream/lake<br>Rainwater<br>Other: Specify
How long does it take to go there, get water and come back?	Number of minutes
Do you collect water for drinking?	Yes<br>No
Are you able to access drinking water at home without assistance?	Yes<br>No
Have you had diarrhea in the past four weeks?	Yes<br>No

Malawi	
Age 2-7 – Caregiver Reported; age 8-17 self reported	
Does [name] have difficulty seeing, even when wearing his/her glasses?	No - no difficulty<br>Yes – some difficulty<br>Yes – a lot of difficulty<br>Cannot do at all
Does [name] have difficulty hearing, even when using his/her hearing aid(s)?	No - no difficulty<br>Yes – some difficulty<br>Yes – a lot of difficulty<br>Cannot do at all
Compared with children of the same age, does [name] have difficulty walking	No - no difficulty<br>Yes – some difficulty<br>Yes – a lot of difficulty<br>Cannot do at all
Age 2-4 Does [name] have difficulty understanding you?	No - no difficulty<br>Yes – some difficulty<br>Yes – a lot of difficulty<br>Cannot do at all
Age 2-4 Do you have difficulty understanding what your child wants?	No - no difficulty<br>Yes – some difficulty<br>Yes – a lot of difficulty<br>Cannot do at all
Age 5+ Compared with children of the same age and using [his/her] usual language, does [name] have difficulty understanding other people	No - no difficulty<br>Yes – some difficulty<br>Yes – a lot of difficulty<br>Cannot do at all
Age 5+ Compared with children of the same age and using [his/her] usual language, does [name] have difficulty being understood by other people?	No - no difficulty<br>Yes – some difficulty<br>Yes – a lot of difficulty<br>Cannot do at all

For peer review only

For peer review only - http://bmjopen.bmj.com/site/about/guidelines.xhtml

Age 5+ Compared with children of the same age, does [name] have difficulty with self-care such as feeding or dressing him/herself?	No - no difficulty<br>Yes – some difficulty<br>Yes – a lot of difficulty<br>Cannot do at all
Age 2-3 Compared with children of the same age, does [name] have difficulty learning the names of common objects?	No - no difficulty<br>Yes – some difficulty<br>Yes – a lot of difficulty<br>Cannot do at all
Age 3+ Compared with children of the same age, does [name] have difficulty learning to do new things?	No - no difficulty<br>Yes – some difficulty<br>Yes – a lot of difficulty<br>Cannot do at all
Age 5+ Compared with children of the same age, does [name] have difficulty remembering things that they have learned?	No - no difficulty<br>Yes – some difficulty<br>Yes – a lot of difficulty<br>Cannot do at all
Age 18+ Individual Self Report if present or household head report if not	
Question	Responses
Do you have difficulty seeing, even if wearing glasses?	No - no difficulty<br>Yes – some difficulty<br>Yes – a lot of difficulty<br>Cannot do at all
Do you have difficulty hearing, even if using a hearing aid?	No - no difficulty<br>Yes – some difficulty<br>Yes – a lot of difficulty<br>Cannot do at all
Do you have difficulty walking or climbing steps?	No - no difficulty<br>Yes – some difficulty<br>Yes – a lot of difficulty<br>Cannot do at all
Do you have difficulty remembering or concentrating?	No - no difficulty<br>Yes – some difficulty<br>Yes – a lot of difficulty<br>Cannot do at all
Do you have difficulty (with self-care such as) washing al over or dressing?	No - no difficulty<br>Yes – some difficulty<br>Yes – a lot of difficulty<br>Cannot do at all
Using their usual (customary) language, does (x) have difficulty communicating, for example: understanding or being understood?	No - no difficulty<br>Yes – some difficulty<br>Yes – a lot of difficulty<br>Cannot do at all
Do you have difficulty raising a 2 liter bottle of water or soda from waist to eye level?	No - no difficulty<br>Yes – some difficulty<br>Yes – a lot of difficulty<br>Cannot do at all
Do you have difficulty using [your/his/her] hands and fingers, such as picking up small objects, for example, a button or pencil, or opening or closing containers or bottles?	No - no difficulty<br>Yes – some difficulty<br>Yes – a lot of difficulty<br>Cannot do at all
Does your household have a latrine?	Yes<br>No
If yes, what type of latrine is it?	Flush to pit

For peer review only - http://bmjopen.bmj.com/site/about/guidelines.xhtml

	Ventilation improved pit latrine<br>Pit latrine without slab<br>Pit latrine without slab/Open pit<br>Other: specify
Do you share this facility with other households?	Used only by your household<br>Shared with other households<br>Public /Communal/ Community latrine
Do you use the same toilet facility as other members of your household	Yes<br>No
If no, why not?	It would be physically impossible<br>I'm not allowed/others would not like it<br>I might face verbal or physical abuse<br>I would be embarrassed<br>Other: specify
Are you usually able to use the toilet facility without you or your clothing coming into contact with faeces or urine	Yes<br>No
What is the main source of drinking water for members of your household?	Piped into dwelling<br>Piped into compound, yard or plot<br>Piped into neighbours<br>Public tap/standpipe<br>Tubewell/borewell<br>Protected well<br>Unprotected well<br>Protected Spring<br>Unprotected Spring<br>Rainwater<br>Tanker Truck<br>Cart with small tank/drum<br>Surface Water<br>Bottled Water<br>Other: Specify
How long does it take for someone to walk to the water source, collect water and come back?	Inside house<br><15 minutes<br>15 – 30 minutes<br>30 minutes – 1 hour<br>>1 hour
Do you collect water for drinking, cooking and other purposes such as for bathing, hand washing and cleaning?	Yes<br>No
If no, why not?	It would be physically impossible<br>I'm not allowed/others would not like it<br>I might face verbal or physical abuse<br>I would be embarrassed<br>Other: specify
Are you able to access drinking water at home without assistance?	Yes<br>No
Has anyone in your household had diarrhea in the past 7 days?	Yes<br>No

For peer review only - http://bmjopen.bmj.com/site/about/guidelines.xhtml

For peer review only

For peer review only - http://bmjopen.bmj.com/site/about/guidelines.xhtml

STROBE Statement—checklist of items that should be included in reports of observational studies

	Item No	Recommendation
**Title and abstract**	1	(*a*) Indicate the study's design with a commonly used term in the title or the abstract **[within Title page and Design section of abstract]**
		(*b*) Provide in the abstract an informative and balanced summary of what was done and what was found **[see results section of abstract p2]**
**Introduction**		
Background/rationale	2	Explain the scientific background and rationale for the investigation being reported **[page 4]**
Objectives	3	State specific objectives, including any prespecified hypotheses **[page 4]**
**Methods**		
Study design	4	Present key elements of study design early in the paper **[page 5]**
Setting	5	Describe the setting, locations, and relevant dates, including periods of recruitment, exposure, follow-up, and data collection **[page 5-7]**
Participants	6	(*a*) *Cohort study*—Give the eligibility criteria, and the sources and methods of selection of participants. Describe methods of follow-up **[N/A]**<br>*Case-control study*—Give the eligibility criteria, and the sources and methods of case ascertainment and control selection. Give the rationale for the choice of cases and controls **[page 5-7]**<br>*Cross-sectional study*—Give the eligibility criteria, and the sources and methods of selection of participants **[page 5-7]**
		(*b*) *Cohort study*—For matched studies, give matching criteria and number of exposed and unexposed **[N/A]**<br>*Case-control study*—For matched studies, give matching criteria and the number of controls per case **[page 5-7]**
Variables	7	Clearly define all outcomes, exposures, predictors, potential confounders, and effect modifiers. Give diagnostic criteria, if applicable **[page 5-7]**
Data sources/ measurement	8*	For each variable of interest, give sources of data and details of methods of assessment (measurement). Describe comparability of assessment methods if there is more than one group **[page 5-7]**
Bias	9	Describe any efforts to address potential sources of bias **[page 7]**
Study size	10	Explain how the study size was arrived at **[page 7]**
Quantitative variables	11	Explain how quantitative variables were handled in the analyses. If applicable, describe which groupings were chosen and why **[page 8]**
Statistical methods	12	(*a*) Describe all statistical methods, including those used to control for confounding **[page 8]**
		(*b*) Describe any methods used to examine subgroups and interactions **[page 8]**
		(*c*) Explain how missing data were addressed **[page 8]**
		(*d*) *Cohort study*—If applicable, explain how loss to follow-up was addressed **[N/A]**<br>*Case-control study*—If applicable, explain how matching of cases and controls was addressed **[page 5-7]**<br>*Cross-sectional study*—If applicable, describe analytical methods taking account of sampling strategy**[page 5-7]**
		(*e*) Describe any sensitivity analyses **[N/A]**

Continued on next page

For peer review only

For peer review only - http://bmjopen.bmj.com/site/about/guidelines.xhtml

**Results**		
Participants	13*	(a) Report numbers of individuals at each stage of study—eg numbers potentially eligible, examined for eligibility, confirmed eligible, included in the study, completing follow-up, and analysed **[page 7, page 9]**
		(b) Give reasons for non-participation at each stage **[page 9]**
		(c) Consider use of a flow diagram **[N/A information in table 2]**
Descriptive data	14*	(a) Give characteristics of study participants (eg demographic, clinical, social) and information on exposures and potential confounders **[Table 2, page 9]**
		(b) Indicate number of participants with missing data for each variable of interest **[provided in relevant tables]**
		(c) *Cohort study*—Summarise follow-up time (eg, average and total amount) **[N/A]**
Outcome data	15*	*Cohort study*—Report numbers of outcome events or summary measures over time **[N/A]**
		Case-control study—Report numbers in each exposure category, or summary measures of exposure **[Table 2, page 9]**
		Cross-sectional study—Report numbers of outcome events or summary measures **[Table 2, 3, 4,5, 6 page 9-15]**
Main results	16	(*a*) Give unadjusted estimates and, if applicable, confounder-adjusted estimates and their precision (eg, 95% confidence interval). Make clear which confounders were adjusted for and why they were included **[Table 2, 3, 4,5,6 page 9-15]**
		(*b*) Report category boundaries when continuous variables were categorized **[Table 2, 3, 4,5,6 page 9-15]**
		(*c*) If relevant, consider translating estimates of relative risk into absolute risk for a meaningful time period **[N/A]**
Other analyses	17	Report other analyses done—eg analyses of subgroups and interactions, and sensitivity analyses **[N/A]**
**Discussion**		
Key results	18	Summarise key results with reference to study objectives **[page 16-17]**
Limitations	19	Discuss limitations of the study, taking into account sources of potential bias or imprecision. Discuss both direction and magnitude of any potential bias **[page 17]**
Interpretation	20	Give a cautious overall interpretation of results considering objectives, limitations, multiplicity of analyses, results from similar studies, and other relevant evidence **[page 17]**
Generalisability	21	Discuss the generalisability (external validity) of the study results **[page 17]**
**Other information**		
Funding	22	Give the source of funding and the role of the funders for the present study and, if applicable, for the original study on which the present article is based **[page 17]**

*Give information separately for cases and controls in case-control studies and, if applicable, for exposed and unexposed groups in cohort and cross-sectional studies.

Note: An Explanation and Elaboration article discusses each checklist item and gives methodological background and published examples of transparent reporting. The STROBE checklist is best used in conjunction with this article (freely available on the Web sites of PLoS Medicine at http://www.plosmedicine.org/, Annals of Internal Medicine at http://www.annals.org/, and Epidemiology at http://www.epidem.com/). Information on the STROBE Initiative is available at www.strobe-statement.org.

For peer review only - http://bmjopen.bmj.com/site/about/guidelines.xhtml

BMJ Open

Access to water and sanitation amongst people with disabilities: results from cross-sectional surveys in Bangladesh, Cameroon, India and Malawi

Journal:	*BMJ Open*
Manuscript ID	bmjopen-2017-020077.R1
Article Type:	Research
Date Submitted by the Author:	02-Feb-2018
Complete List of Authors:	Mactaggart, Islay; London School of Hygiene and Tropical Medicine,<br>Schmidt, Wolf-Peter; London School of Hygiene & Tropical Medicine,<br>Bostoen, Kristof; IRC<br>Chunga, Joseph; Centre for Social Research, University of Malawi,<br>Danquah, Lisa; Oxford University, Centre for Tropical Medicine and Global Health<br>Halder, Amal; icddr,b, Environmental Interventions Unit<br>Parveen Jolly, Saira; Research and Evaluation Division, BRAC<br>Polack, Sarah; London School of Hygiene & Tropical Medicine, Dept of Clinical Research<br>Rahman , Mahfuzar ; Research and Evaluation Division, BRAC,<br>Snel, Marielle; World Vision International<br>Kuper, Hannah; The London School of Hygiene & Tropical Medicine, Clinical Research<br>Biran, Adam; London School of Hygiene and Tropical Medicine, International Centre for Evidence in Disability
**Primary Subject Heading**:	Global health
Secondary Subject Heading:	Epidemiology
Keywords:	disability, population-based surveys, water, sanitation and hygiene

SCHOLARONE™
Manuscripts

Access to water and sanitation amongst people with disabilities: results from cross-sectional surveys in Bangladesh, Cameroon, India and Malawi

Authors: Islay Mactaggart[1], Wolf-Peter Schmidt[2], Kristof Bostoen[3], Joseph Chunga[4], Lisa Danquah[5], Amal Krishna Halder[6], Saira Parveen Jolly[7], Sarah Polack[1], Mahfuzar Rahman[7], Marielle Snel[8], Hannah Kuper[1], Adam Biran[2]

Corresponding author:

Ms. Islay Mactaggart

LSHTM

Keppel Street, London, WC1E 7HT, UK

E: Islay.mactaggart@lshtm.ac.uk

T: +44 (0) 2079588334

Affiliations:

[1]International Centre for Evidence in Disability, London School of Hygiene & Tropical Medicine, London UK

[2]Environmental Health Group, London School of Hygiene & Tropical Medicine, London UK

[3]Monitoring[4] chΔnge and IRC-Associate, the Hague Netherlands

[4]Centre for Social Research, University of Malawi, Zombo Malawi

[5] Centre for Tropical Medicine and Global Health, Oxford University, UK

[6]icddr,b, Dhaka Bangladesh

[7]Resarch and Evaluation Division, BRAC, Dhaka Bangladesh

[8] World Vision International, Geneva Switzerland

For peer review only - http://bmjopen.bmj.com/site/about/guidelines.xhtml

ABSTRACT

Objectives: To assess access to adequate Water, Sanitation and Hygiene (WASH) among people with disabilities at the household and individual level.

Design: Cross-sectional surveys.

Setting: Data were included from five district- or regional-level surveys: two in Bangladesh (Bangladesh-1, Bangladesh-2), and one each in Cameroon, Malawi, and India.

Participants: 99,252 participants were sampled across the datasets (range: 3567 - 75,767), including 2494 with disabilities (93 –1374).

Outcome: Prevalence of access to WASH at household and individual level.

Data analysis: Age/sex disaggregated disability prevalence estimates were calculated accounting for survey design. The UNICEF/WHO Joint Monitoring Programme definitions were used to classify facilities as improved/unimproved. Multivariable logistic regression was undertaken to compare between households with/without a person with a disability, and to identify predictors of access amongst people with disabilities.

Results: There were no differences in access to improved sanitation or water sources between households with/without members with disabilities across the datasets. In Bangladesh-2, households including a person with a disability were more likely to share facilities with other households (Odds Ratio [OR] =1.3, 95% Confidence Interval [95% CI] 1.1 – 1.5). Households with people with disabilities were more likely to spend > 30 minutes (round-trip) collecting drinking water than households without in both Cameroon (OR=1.8, 1.0 – 3.4) and India (OR=2.3, 1.2 – 4.7). Within households, people with disabilities reported difficulties collecting water themselves (23% - 80% unable to) and accessing the same sanitation facilities as other household members, particularly without coming into contact with faeces (up to 47% in Bangladesh-2). These difficulties were most marked for people with more severe impairments.

Conclusions: People with disabilities may not have poorer access to WASH at the household level, but may have poorer quality of access within their households. Further programmatic work is needed to ensure WASH facilities are inclusive of people with disabilities.

For peer review only - http://bmjopen.bmj.com/site/about/guidelines.xhtml

Strengths and limitations of this study

- The surveys were relatively large and conducted in geographically different areas.
- Detailed information was collected on access to WASH and the presence of disabilities in different settings.
- Different methods and disability definitions were used across the surveys, making comparison of prevalence difficult.
- Each survey was conducted at the state or regional level and the estimates derived therefore do not constitute national estimates.
- Comparative data on indicators of the quality of access to WASH amongst people without disabilities were not collected in any of the studies (e.g. could hygiene facilities be used without incurring pain).

For peer review only

For peer review only - http://bmjopen.bmj.com/site/about/guidelines.xhtml

INTRODUCTION

Access to clean water, adequate sanitation and hygiene (WASH) for the prevention or mitigation of faeco-oral infections is a fundamental component of public health[1-4]. This is underlined by the focus of Sustainable Development Goal 6: Ensure access to water and sanitation for all[5]. 2015 estimates suggest that 91% of the global population now use an improved drinking water source (protected from outside contamination) and 68% use an improved sanitation facility (one that separates human excreta from human contact), with 1.5% of the current global disease burden attributed to unimproved water and sanitation [2 6-8]. However, these aggregate global figures may mask vulnerabilities within specific populations [9 10].

Persons with disabilities are described in the United Nations Convention on the Rights of Persons with Disabilities (UNCRPD) as those who “experience long-term physical, mental, intellectual or sensory impairments which, in interaction with various barriers, may hinder their full and effective participation in society on an equal basis with others” [11]. Persons with disabilities constitute up to 15% of the global population, and are asserted to experience poorer access to WASH, although few quantitative data exist [12]. These assertions are grounded in the limited, but growing, body of evidence that associates disability status with greater risk of widespread exclusion from education and economic productivity, alongside poor access to health care in multiple countries and contexts [12-14]. A review by Groce et al. (2011) lists potential barriers to WASH amongst persons with disabilities in the spheres of technical access barriers (such as facility structure and distance to facilities), social barriers related to stigma or abuse, and communication barriers [15]; all supported by qualitative research [16]. The presence of social barriers, alongside the interrelationships between WASH and poverty, and poverty and disability, may also impact on WASH access within the household. Many censuses and household surveys have collected data on both WASH access and disability, however, few surveys have reported on the association. WASH access is usually only measured at the household level, and existing studies have used inconsistent definitions of disability. Quantitative data therefore remain lacking on whether persons with disabilities live in households that have poorer access to WASH, and whether persons with disabilities have poorer WASH access than other members of their household.

To address these gaps in knowledge, we analysed data from five cross-sectional, population-based studies conducted at the district or regional level that had collected data on disability and access to, and experience of, WASH. These studies were conducted in: Cameroon, Malawi, India, and Bangladesh (two discrete studies). This secondary analysis is opportunistic, based on data available. However, the inclusion of data from two African and two South-East Asian settings allows a focus on the regions with lowest access to improved facilities globally[17].

We explored 1) whether households including persons with disabilities have different access to WASH compared to households without disabled members; 2) whether persons with disabilities have different access to WASH compared to other members of their household; and 3) which factors predict access to WASH among persons with disabilities.

For peer review only - http://bmjopen.bmj.com/site/about/guidelines.xhtml

METHODS

We analysed data from five cross-sectional, population-based studies conducted at the district or regional level that had collected data on disability and access to WASH. These studies were conducted between 2013 - 2015 in: Cameroon, Malawi, India, and Bangladesh (two discrete studies).

Table 1 provides a comparative overview of the datasets in each study, including definitions of disability and WASH used.

Each of the studies comprised a single- or multiple- district, population-based survey. One of the studies conducted a census, that is, included all individuals living within the defined geographical area (Bangladesh-2). The remaining four studies utilized cluster sampling to select a random sample of participants. The samples were not nationally representative and are referred to by their country of origin for brevity. In all settings, a household-level questionnaire was administered and additional questionnaire modules were used for people identified through the survey as having disabilities. In Cameroon, India and Malawi, a nested case-control study was undertaken, whereby cases with disabilities and age-sex-cluster-matched controls without disabilities were selected for further structured interview. In all settings written informed consent was collected before the questionnaire was administered, and ethical approval was obtained from the relevant boards. Ethical Committees that approved each study are provided in Supplementary File 1[18].

Disability assessment

Four of the five surveys (Bangladesh-2, Cameroon, India and Malawi) used iterations of the Washington Group Short Set (WGSS) questions to screen for disability in the population (see Appendix 1 for full question sets). [19 20]. This questionnaire assesses disability status via reported limitation in core functional domains (such as seeing, hearing and self-care) on a 4-point scale ("no difficulty", "some difficulty", "a lot of difficulty" and "cannot do at all"). Participants reporting "a lot of difficulty" or "cannot do at all" for any item in the questionnaire were considered to have a disability in each of the four studies. In Cameroon and India, the Washington Group screen was supplemented with clinical screening to detect the presence of moderate or severe physical, visual or hearing impairments or severe health conditions (epilepsy and depression) using pre-validated screening methods and thresholds [21-24]. Conversely, Bangladesh-1 used a binary screen for disability whereby disability status was reported by the household head for each member of their household as "Disability – Yes or No". If the household head reported that a member of their household had a disability, they were asked to identify the type of disability (e.g. vision, hearing). Type of disability in Bangladesh-1 also included "age-related", defined as related to the process of ageing.

WASH assessment

The WASH question sets used in each study were adaptations of the World Health Organisation/ United Nations Children's Fund Joint Monitoring Programme (JMP) *Core Questions on Drinking Water and Sanitation for Household Surveys* and the JMP draft intra-household Question Set (see Appendix 1 for full question sets) [25 26]. Information about WASH at the household level was

For peer review only - http://bmjopen.bmj.com/site/about/guidelines.xhtml

collected through interview with either the head of household (Bangladesh-1, Bangladesh-2, Malawi) or with people with disabilities and matched control subjects (Cameroon, India) as follows:

- Type of sanitation facility
- Ownership of facility
- Drinking water source (excluded in Bangladesh-1)
- Time to reach water point, collect water and return to household (excluded in Bangladesh-1)
- Recent episodes of diarrhoea (Malawi: in last 7 days; India/Cameroon: previous four weeks; Bangladesh-1 and Bangladesh-2 excluded).

Individual-level WASH access data were collected from all participants identified to have a disability in the survey, and via a proxy (usually their primary caregiver or the head of the household) for those who were not able to communicate independently (except Bangladesh-2). Individual data were not available for the Bangladesh-2 study. Across the other data sets the following data were collected from individuals:

- Use of the same sanitation facility as other household members
- Reasons if a different facility is used
- Whether the facility can be accessed without contact with faeces
- Whether drinking water can be collected by the individual
- Reasons if drinking water cannot be collected by the individual
- Whether water can be accessed within the household without assistance
- Whether the same bathing place is used as other household members

Other data collected

In each survey the household head (Bangladesh-1, Bangladesh-2, Malawi) or participants with disabilities and matched control subjects (Cameroon, India) reported on the demographic composition of the household and provided data on socio-economic status (e.g. asset ownership, material of the roof/floor/wall, income).

For peer review only

For peer review only - http://bmjopen.bmj.com/site/about/guidelines.xhtml

Table 1: Study Methods					
	**Bangladesh-1 (icddr,b)**	**Bangladesh-2 (IRC-BRAC)**	**Cameroon (ICED, LSHTM)**	**India (ICED, LSHTM)**	**Malawi (collaboration[1])**
**Geographical Location**	177 sub-districts (upazillas) across Bangladesh (est. population size 750, 000)	Rangpur and Rajshahi Districts, Bangladesh (est. population size 34,149,858)	Fundong Health District, North-West Region (est. population size 125,604)	Mahbubnager District, Telegana State (est. population size 4,053,028)	Rumphi District, Northern Region (est. population size 203,054)
**Year of data collection**	2014	2015	2013	2014	2014
**Sample size (response rate %)**	1,248 households (90%)	20,000 households (98%)	4,080 individuals (87%)	4,080 individuals (88%)	1,800 households (100%)
**Sampling strategy**	Two-stage Cluster Randomised Sampling with Probability Proportionate to Size, stratified by household wealth category	Population census	Two-stage Cluster Randomised Sampling with Probability Proportionate to Size, stratified by urban and rural	Two-stage Cluster Randomised Sampling with Probability Proportionate to Size, stratified by urban and rural	Two-stage Cluster Randomised Sampling across 4 purposively selected administration units[2]
**Study definition of disability Status**	Yes/No reported by household head followed by list of conditions	Washington Group short set reported by individual if present, or female household head if not	Washington Group short set+2 criteria (Self report unless <8) or clinical impairment	Washington Group short set+2 (Self report unless <8) or clinical impairment	Washington Group extended set (report by household head or primary caregiver)
**Population-level WASH Data**	*Yes – sanitation only*	*Yes*	*No –data for case and control households only*	*No –data for case and control households only*	*Yes*
**Household-level WASH data**	*Yes – sanitation only*	*Yes*	*Yes – data for case and control households only*	*Yes – data for case and control households only*	*Yes*
**Individual level WASH data**	*Yes – people with disabilities only*	*Yes – sanitation only*	*Yes – people with disabilities only*	*Yes – people with disabilities only*	*Yes – people with disabilities only*
[1] Environmental Health Group (EHG) at the London School of Hygiene & Tropical Medicine (LSHTM), the Water Engineering and Development Centre (WEDC) at Loughborough					

For peer review only

University, Mzuzu University and theCentre for Social Research at the University of Malawi; [2]Purposive selection criteria: limited exposure to sanitation promotion and relative ease of access

For peer review only

For peer review only - http://bmjopen.bmj.com/site/about/guidelines.xhtml

Data Analysis

Data were analysed in Stata 12.0[27]. Descriptive statistics were used to describe each dataset.

Disability prevalence estimates for each study area were determined using the svy command to account for clustering in each of the survey designs. Severity of disability was calculated for all datasets except Bangladesh-1 (which did not include a measure of severity) as follows:

- Moderate: those with moderate clinical impairments or reporting maximum "a lot of difficulty" in at least one domain of the WGSS.
- Severe: those with severe clinical impairments or reporting "cannot do" in at least one domain of the WGSS.

Socio-economic status (SES) indices were created for Bangladesh-1, Cameroon and India using principal component analysis of assets, and divided into quartiles[28]. Bangladesh-2 data were pre-coded by interviewers at the data collection stage as ultra-poor, poor and non-poor based on land ownership and livelihood category[29]. In Malawi, annual average household income was collected as a continuous variable and divided into quartiles.

Using the JMP Millennium Development definitions, household water and sanitation facilities were classified as improved or unimproved as follows:[7]

- Improved sanitation: connected to public or private sewer; connected to septic system; pour-flush latrine; simple pit latrine; ventilated improved pit latrine
- Unimproved sanitation: public or shared latrine; open pit latrine; bucket latrine
- Improved water source: piped into household; public standpipe; borehole; protected dug well; protected spring; rainwater collection
- Unimproved water source: unprotected well, unprotected spring; rivers or ponds; vendor-provided water; bottled water; tanker truck water

We assessed whether household-level access to WASH was different in households with a disabled member compared to households that included no disabled member using multivariable logistic regression. In Malawi and Bangladesh-2 these data were available for all households. In Cameroon and India, these data were available for all households that included a person with a disability, and for age-sex-cluster matched control households that did not include a person with a disability. Household-level water access data were not available for the Bangladesh-1 study.

We evaluated whether individuals with disabilities have poorer WASH access than other members of their household by describing the reported difficulties of people with disabilities in accessing the same WASH facilities as other household members (data not available for Bangladesh-2 study). Furthermore, predictors (e.g. type of impairment, age or gender) of good intra-household access to WASH among people with disabilities were explored using multivariable logistic regression for the Cameroon, India and Malawi studies.

Missing data were excluded from analyses and listed as such in relevant tables.

For peer review only - http://bmjopen.bmj.com/site/about/guidelines.xhtml

RESULTS

A total of 99,254 individuals in 24,189 households were sampled across the five datasets (ranging between 3567 and 75,767 per dataset). The age and gender distribution of the datasets are given in Table 2. In all, the population was relatively evenly distributed between males and females, and a large proportion of the sample were children or young adults. The household size ranged from an average of 3.9 in Bangladesh-2 to 7.8 in Cameroon.

Table 2: Study sample characteristics

	BGD-1	BGD-2	Cameroon	India	Malawi
**Sample size - Households**	1207	19, 627	677	875	1803
**Sample size – individuals**	7511	75767	3467	3573	8834
**Gender**					
Female	3867 (51.5%)	37,076 (48.9%)	2112 (59.2%)	1866 (52.2%)	4500 (50.9%)
**Age[a]**					
0-17	2802 (37.3%)	26,392 (34.8%)	1950 (54.7%)	1223 (34.2%)	4562 (51.6%)
18-24	909 (12.1%)	49,375 (65.2%)	317 (8.9%)	473 (13.2%)	1179 (13.3%)
25-44	2090 (27.8%)	-	572 (16.0%)	986 (27.6%)	1862 (21.1%)
45-64	1204 (16.0%)	-	396 (11.1%)	689 (19.3%)	826 (9.4%)
65+	506 (6.7%)	-	332 (9.3%)	202 (5.7%)	405 (4.6%)
**Mean household size**	6.2 (SD 3.4)	3.9 (SD 1.5)	7.8 (SD 4.8)	5.2 (SD 2.8)	4.9 (SD 2.1)

[a]Age categories restricted to 0-1, 2-9, 10-19 and 20+ in Bangladesh-2 raw data, therefore Bangladesh-2 age data refer to 0-19 and 20+

Socio-economic status did not differ between households with and without a person with a disability in Bangladesh-1, Bangladesh-2, India and Malawi (data not shown). In Cameroon, mean asset scores were significantly lower for households with people with disabilities (-0.39, SD 2.4) than amongst households without people with disabilities (0.37, 2.6).

There was a broad range in the prevalence of disability across the settings: 1.3% (95% CI 1.0 - 1.7) in Bangladesh-1; 1.9% (1.6 – 2.1) in Bangladesh-2; 2.6% (2.4 – 2.8) in Malawi; 11.0% (9.4 – 12.7) in Cameroon and 12.6% (10.9 – 14.5) in India (Table 3). Four studies used iterations of the Washington Group Questions, supplemented by clinical tools in Cameroon and India. Disability prevalence restricted to the Washington Group Short Set only was similar across settings: 1.9% (1.6 – 2.1) in Bangladesh-2; 2.6% (2.4 – 2.8) in Malawi; 11.0% (9.4 – 12.7); 5.8% (4.6 – 7.4) in Cameroon and 7.1% (5.7 – 8.9) in India.

In each study, prevalence was similar between men and women, and increased strongly with age. The most frequent type of functional limitation reported was physical (walking or climbing stairs) in each of the five datasets. The proportions of people with disabilities reporting other functional difficulties (hearing, visual or intellectual) varied across the surveys, but were overall

For peer review only - http://bmjopen.bmj.com/site/about/guidelines.xhtml

broadly similar in four of the five datasets. Bangladesh-1 included "age-related" as a type of disability, which comprised 21.5% of the distribution.

The proportion of households including one or more persons with disabilities varied substantially between 6.5% in Bangladesh-2 and 39.3% in Cameroon.

In Bangladesh-1 and Bangladesh-2, WASH access was compared between all households either with or without a person with a disability (as identified in the population-based survey). In Cameroon, India and Malawi, comparison was made between households with a person with a disability and households with age, gender and cluster matched controls only. Table 4 provides the sample characteristics for these nested case-control studies. The nested case-control studies are therefore smaller in sample size than the two Bangladesh comparative survey samples.

Table 5 presents access to WASH amongst households with and without persons with disabilities in each dataset. No statistically significant differences in access to an improved sanitation facility were observed between households with and without a person with a disability in any of the five datasets. In Bangladesh-2, households with a person with a disability were more likely to share their sanitation facility with another household than were households having no member with a disability (Odds Ratio=1.3, 95% Confidence Interval 1.1 – 1.5) but there were no other differences in household sanitation facility ownership across datasets.

There was no difference in prevalence of access to an improved water source between households with and those without a member with a disability in any of the datasets. In Bangladesh-2 and Malawi, there was also no observed relationship between the length of time taken to collect water and disability presence in the household. However, households with a person with a disability were more likely to spend more than 30 minutes fetching household water than households without a disabled member in both Cameroon (OR=1.9, 1.1 – 3.5) and India (OR=2.4, 1.2 – 4.8). No differences in frequency of reported diarrhoeal episodes were observed by individual disability status in Cameroon or India (Cameroon OR=1.2, 0.8 – 1.9; India 0.9, 0.6 – 1.4), or household disability status in Malawi (0.7, 0.4 – 1.2).

For peer review only - http://bmjopen.bmj.com/site/about/guidelines.xhtml

Table 3 – Prevalence of disability in the 5 datasets (%, 95% CI)[a]

	BGD-1	BGD-2	Cameroon	India	Malawi
**Sample size (individuals)**	**7511**	**75767**	**3467**	**3573**	**8834**
**Overall prevalence of disability[a]**	1.3 (1.0 – 1.7)	1.9 (1.6 – 2.1)	11.0 (9.4 – 12.7)	12.6 (10.9 – 14.5)	2.6 (2.4 – 2.8)
**WGSS prevalence of disability**	-	1.9 (1.6 – 2.1)	5.8 (4.6 – 7.4)	7.1 (5.7 – 8.9)	2.6 (2.4 – 2.8)
**Gender**					
- Male	1.3 (1.0 – 1.8)	2.0 (1.7 – 2.3)	10.6 (8.9 – 12.5)	12.0 (9.9 – 14.4)	2.5 (2.1 – 3.1)
- Female	1.3 (0.9 – 1.9)	1.8 (1.5 – 2.0)	11.2 (9.3 – 13.5)	13.2 (11.3 – 15.3)	2.7 (2.3 – 3.1)
**Age**					
- 2-17	0.5 (0.3 – 0.9)	1.1 (0.9 – 1.2)	4.9 (3.9 – 6.1)	3.8 (2.7 – 5.3)	1.2 (1.0 – 1.5)
- 18-49	0.5 (0.3 – 0.9)	2.3 (1.9 – 2.6)[c]	6.9 (5.3 – 9.1)	8.2 (6.0 – 11.0)	1.6 (1.3 – 2.1)
- 50+	4.7 (3.4 – 6.5)	-	33.7 (28.8 – 38.9)	38.3 (33.6 – 43.3)	11.5 (10.2 – 12.9)
**Type of functional difficulty[b]**					
- Hearing	9.7 (4.5 – 17.6)	21.1 (19.0 – 23.4)	38.3 (33.2 – 43.5)	39.3 (34.6 – 44.0)	29.0 (23.1 – 35.6)
- Visual	6.5 (2.4 – 13.5)	26.5 (24.2 -28.9)	31.4 (26.6 – 36.5)	37.8 (33.2 – 42.5)	39.2 (32.6 – 40.0)
- Physical	41.9 (31.8 – 52.6)	57.6 (55.0 – 60.3)	53.3 (48.0 – 58.5)	51.7 (46.9 – 56.5)	50.2 (43.4 – 57.1)
- Intellectual	20.4 (12.8 - 30.1)	27.4 (25.0 – 29.8)	20.2 (16.2 – 24.7)	36.8 (21.2 – 59.0)	25.3 (19.7 – 31.7)
- Age-Related	21.5 (13.7 – 31.2)	-	-	-	-
**Proportion households with at least 1 person with a disability**	7.1(5.6 – 9.0)	6.5 (5.7 – 7.5)	39.3 (34.0 – 44.7)	38.5 (34.1 – 43.1)	10.9 (9.5 – 12.4)

[a] Disability prevalence data restricted to population aged 2+ years only

[b] Functional difficulty reported amongst people classified as having a disability. These figures are not prevalence estimates and the categories are not mutually exclusive.

[c] Age categories restricted to 0-1, 2-9, 10-19 and 20+ years in Bangladesh-2 raw data therefore Bangladesh-2 age data refer to 0-19 and 20+

For peer review only

Table 4: Nested Case-Control Study Sample Characteristics in Cameroon, India and Malawi						
	Cameroon		India		Malawi	
	No Disability	Disability	No Disability	Disability	No Disability[a]	Disability
**Sample size (individuals)**	274	429	337	508	200	215
	N (%)	N (%)	N (%)	N (%)	N (%)	N (%)
**Age group**						
- 5-17	90 (33%)	114 (27%)	49 (15%)	67 (13%)	-	47 (22%)
- 18-49	87 (32%)	87 (20%)	160 (48%)	177 (35%)	142 (71%)	54 (25%)
- 50+	97 (35%)	228 (53%)	128 (38%)	264 (52%)	58 (29%)	113 (53%)
**Sex**						
- Male	113 (41%)	179 (42%)	163 (48%)	236 (46%)	158 (79%)	101 (47%)
- Female	161 (59%)	250 (58%)	174 (52%)	272 (54%)	42 (21%)	113 (53%)
**SES Quartile**						
- 1st Quartile (poorest)	64 (23%)	104 (24%)	67 (20%)	142 (28%)	29 (15%)	51 (24%)
- 2nd Quartile	56 (21%)	117 (27%)	87 (26%)	120 (24%)	81 (41%)	79 (37%)
- 3rd Quartile	61 (22%)	105 (24%)	94 (28%)	124 (24%)	43 (22%)	37 (17%)
- 4th Quartile (richest)	92 (34%)	103 (24%)	89 (26%)	121 (24%)	47 (24%)	46 (21%)
**Disability Type[a]**						
- Hearing	-	142 (33%)	-	189 (37%)	-	61 (29%)
- Visual	-	123 (29%)	-	176 (35%)	-	81 (48%)
- Physical	-	257 (58%)	-	296 (58%)	-	101 (47%)
- Intellectual	-	119 (28%)	-	38 (7%)	-	51 (24%)
[a]Household Head details						

For peer review only - http://bmjopen.bmj.com/site/about/guidelines.xhtml

Access to WASH among people with disabilities in comparison to their household members without disabilities is shown in Table 6. The majority of people with disabilities reported that they were able to access the same sanitation facility as other members of their household (including those households without access to any facility). Amongst those who could not, physical access issues were most commonly reported. A substantial proportion of people with disabilities were not able to access sanitation facilities without either themselves or their clothing coming into contact with faeces. Few people with disabilities in Bangladesh-1 were able to collect water from the source themselves (21%), though the sample size was relatively small (n=78), while this proportion was higher in the other surveys (52% in Malawi to 77% in India). Physical barriers and disapproval from others were the most commonly reported reasons for persons with disabilities not collecting water themselves across the datasets. Most people with disabilities reported that they could access drinking water in the house without assistance and could use the same bathing place as other household members.

Table 7 presents the correlates of intra-household WASH access amongst people with disabilities for the three datasets that included these data (Cameroon, India and Malawi). People with more severe disabilities generally had poorer WASH access (for example a much lower likelihood [OR=0.1, 95% CI 0.1 – 0.3] of using the same sanitation facility as other household members, or using the facility without coming into contact with faeces [0.2, 0.1 – 0.3]) than people with less severe disabilities. Furthermore, older people with disabilities were more likely than children with disabilities to have access to the same sanitation facilities as other household members in all three settings. There was evidence that among people with disabilities, women were more likely than men to use the same sanitation facilities as other members of the household in Malawi, and to collect water for themselves in Cameroon and Malawi.

For peer review only - http://bmjopen.bmj.com/site/about/guidelines.xhtml

Table 5: Access to WASH amongst households with and without people with disabilities

	BGD-1			BGD-2			Cameroon			India			Malawi		
	No disability n=1121	*Disability n=86*	OR (95% CI)	*No disability n=18364*	*Disability n=1284*	OR (95% CI)	*No disability n=274*	*Disability n=429*	OR (95% CI)	*No disability n=337*	*Disability n=508*	OR (95% CI)	*No disability n=200*	*Disability n=215*	OR (95% CI)
**Sanitation facility**															
Unimproved	34.5%	31.4%	Ref.	17.4%	20.3%	Ref.	92.4%	91.4%	Ref.	23.4%	23.3%	Ref.	99.5%	99.5%	Ref.
Improved	65.5%	68.6%	1.1 (0.7 – 1.8)	82.7%	79.8%	0.8 (0.7 – 1.0)	7.6%	8.6%	0.9 (0.5 – 1.6)	76.6%	76.7%	1.0 (0.7 – 1.4)	0.5%	0.5%	1.1 (0.1- 17.3)
**Ownership**															
Used only by hh	82.2%	81.4%	Ref.	68.9%	63.0%	Ref.	86.0%	87%	Ref.	52.9%	53.3%	Ref.	-	-	
Shared ownership w/ other households	8.7%	10.5%	1.2 (0.6 – 2.5)	30.2%	35.8%	1.3 (1.1 – 1.5)*	14.0%	13.0%	0.9 (0.6 – 1.4)	14.4%	13.5%	0.9 (0.6 – 1.4)	-	-	-
Public	9.1%	8.1%	0.9 (0.4 – 2.0)	0.1%	0.1%	2.6 (0.3 – 21.6)	0	0.2%	-	32.7%	33.2%	1.0 (0.7 – 1.4)	-	-	
Don't Know	-	-	-	0.9%	1.2%	1.4 (0.8 – 2.4)	0	0	-	0	0	-	-	-	
**Time to water source**[a]															
≤ 30 m.	-	-	-	0%	0%	-	94.2%	89.6%	Ref.	96.6%	92.1%	Ref.	86.5%	80.9%	Ref.
> 30 m.				100%	100%		5.8%	10.4%	1.9 (1.1 – 3.5)*	3.4%	7.9%	2.4 (1.2 – 4.8)*	13.5%	19.1%	1.5 (0.9 – 2.6)
**Water source**															
Unimproved	-	-	-	0%	0%	-	39.2%	37.3%	Ref.	23.4%	23.3%	Ref.	12.5%	11.6%	Ref.
Improved				100%	100%		60.8%	62.7%	1.1 (0.8 – 1.5)	76.6%	76.7%	1.0 (0.7 – 1.4)	87.5%	88.4%	1.1 (0.6 – 2.0)
**At least one episode of diarrhoea in the last four weeks**[b]	-	-	-	-	-	-	11.7%	13.8%	1.2 (0.8 – 1.9)	12.8%	11.8%	0.9 (0.6 – 1.4)	21.5%	17.2%	0.8 (0.5 – 1.3)

*denotes statistical significance at the 95% confidence level

[a] Round trip travel time

[b] Refers to any episode of diarrhoea in the household on a seven day recall-period in Malawi; and any episode of diarrhoea experienced by the individual respondent on a four week recall-period in India and Cameroon

For peer review only

Table 6 – Access to WASH amongst individuals with disabilities							
	**BGD-1**	**BGD-2**	**Cameroon**	**India**	**India – Open Def. only**	**Malawi**	**Malawi – Open Def. only**
	*n=78**	*n=1374*	*n=429*	*n=508*	*n=274*	*n=215*	*n=36*
**Use same sanitation facility as other household members**	81%	82.0%	90%	95%	94%	94.4%	89%
**Reasons for using a different facility[a]**							
Physically impossible	93%		73%	28%	19%	33%	43%
Others would not like it/not allowed			5%	0	0	17%	0
Physical or verbal abuse		-	0	0	0	17%	0
I would be embarrassed			0	0	0	8%	0
Other	7%		23%	72%	81%	25%	57%
**Can access sanitation facility without contact with faeces**	53%	-	86%	58%	55%	86%	83%
**Can collect drinking water themselves**	21%	-	69%	77%	-	53%	67%
**Reasons cannot collect drinking water themselves**							
Physically impossible	50%		80%	86%		74%	
Others would not like it/not allowed	50%		13%	5%	-	8%	-
Physical or verbal abuse	0		0	0		3%	
I would be embarrassed	0		0	0		5%	
Other	0		8%	9%		10%	
**Can access drinking water at home without assistance**	88%[a]	-	86.%[a]	59%[a]	-	84%	81%
**Use same Bathing Source as other household members**	81%[a]	-	98%[a]	97%[a]	-	95%	86%

*8 households with a confirmed member with a disability excluded due to missing data

[a]This is of amongst the proportion who state they use a different facility

[b]This is of amongst the proportion who state they cannot collect water themselves

[c] This is amongst the proportion that collect water themselves

For peer review only

Table 7 – Age and sex adjusted correlates of WASH access amongst people with disabilities

		Same sanitation facility as other household members			Access without contact with faeces			Collect water for themselves		
		No n (%)	Yes n (%)	Age and Sex Adj OR (95% CI)	No n (%)	Yes n (%)	Age and Sex Adj OR (95% CI)	No n (%)	Yes n (%)	Age and Sex Adj OR (95% CI)
Cameroon (n=412)	Age group									
	5 – 17	18 (45.0)	93 (25.0)	Ref.	24 (40.7)	87 (24.7)	Ref.	26 (20.2)	85 (30.0)	Ref.
	18-49	5 (12.5)	81 (21.8)	2.8 (1.0 –8.0)	11 (18.6)	75 (21.3)	1.8 (0.8 – 4.0)	19 (14.7)	67 (23.7)	1.0 (0.5 – 1.9)
	50+	17 (42.5)	198 (53.2)	2.1 (1.0 – 4.3)	24 (40.7)	191 (54.1)	2.1 (1.2 – 4.0)*	84 (65.1)	131 (46.3)	0.4 (0.3 – 0.7)*
	Gender									
	Male	24 (60.0)	150 (40.3)	Ref.	29 (49.2)	145 (41.1)	Ref.	70 (54.3)	104 (36.8)	Ref.
	Female	16 (40.0)	222 (59.7)	2.0 (1.0 – 4.0)	30 (50.9)	208 (58.9)	1.3 (0.7 – 2.3)	59 (45.7)	179 (63.3)	2.1 (1.4 – 3.3)*
	Type									
	Multiple	23 (57.5)	126 (33.9)	0.6 (0.3 – 1.3)	29 (49.2)	120 (34.0)	0.8 (0.4 – 1.5)	62 (48.1)	87 (30.7)	0.6 (0.4 – 1.1)
	Hearing	2 (5.0)	49 (13.2)	2.3 (0.5 – 10.9)	5 (8.5)	46 (13.0)	1.5 (0.5 – 4.3)	13 (10.1)	38 (13.4)	1.6 (0.8 – 3.5)
	Seeing	2 (5.0)	49 (13.2)	2.9 (0.6 – 13.8)	3 (5.1)	48 (13.6)	3.2 (0.9 – 11.3)	12 (9.3)	39 (13.8)	1.8 (0.8 – 4.0)
	Intellectual	0 (0.0)	27 (7.3)	-	0 (0.0)	27 (7.7)	-	2 (1.6)	25 (8.8)	4.5 (1.0 – 20.7)
	Physical	13 (32.5)	121 (32.5)	Ref.	22 (37.3)	112 (31.7)	Ref.	40 (31.0)	94 (33.2)	Ref.
	Severity									
	Moderate	19 (47.5)	324 (87.1)	Ref.	32 (54.2)	311 (88.1)	Ref.	89 (69.0)	254 (89.8)	Ref.
	Severe	21 (52.5)	48 (12.9)	0.1 (0.1 – 0.3)*	27 (45.8)	42 (11.9)	0.2 (0.1 – 0.3)*	40 (31.0)	29 (10.3)	0.2 (0.1 – 0.3)*
India (n=508)	Age group									
	5 – 17	9 (36.0)	58 (12.0)	Ref.	28 (13.0)	39 (13.3)	Ref.	18 (15.4)	49 (12.5)	Ref.
	18-49	11 (44.0)	166 (34.4)	2.4 (0.9 – 6.0)	75 (34.9)	102 (34.8)	1.0 (0.6 – 1.7)	27 (23.1)	150 (38.4)	2.0 (1.0 – 4.0)
	50+	5 (20.0)	259 (53.6)	8.2 (2.6 – 25.4)*	112 (52.1)	152 (51.9)	1.0 (0.6 – 1.7)	72 (61.5)	192 (49.1)	1.0 (0.5 – 1.8)
	Gender									
	Male	10 (40.0)	231 (47.8)	Ref.	98 (45.6)	143 (48.8)	Ref.	58 (49.6)	183 (46.8)	Ref.
	Female	15 (60.0)	252 (52.2)	0.7 (0.3 – 1.6)	117 (54.4)	150 (51.2)	0.9 (0.6 – 1.3)	59 (50.4)	208 (53.2)	1.1 (0.7 – 1.7)
	Type									
	Multiple	13 (52.0)	186 (38.5)	0.8 (0.3 – 2.0)	91 (42.3)	108 (36.9)	0.8 (0.5 – 1.3)	57 (48.7)	142 (36.3)	1.0 (0.6 – 1.8)
	Hearing	1 (4.0)	78 (16.2)	5.4 (0.7 – 42.7)	26 (12.1)	53 (18.1)	1.5 (0.8 – 2.5)	14 (12.0)	65 (16.6)	2.3 (1.1 – 4.6)*
	Seeing	0 (0.0)	76 (15.7)	-	35 (16.3)	41 (14.0)	0.8 (0.4 – 1.4)	9 (7.7)	67 (17.1)	3.4 (1.5 – 7.6)*
	Intellectual	1 (4.0)	16 (3.3)	0.8 (0.1 – 7.7)	7 (3.3)	10 (3.4)	2.1 (0.4 – 11.0)	4 (3.4)	13 (3.3)	0.9 (0.2 – 5.0)
	Physical	10 (40.0)	127 (26.3)	Ref.	56 (26.1)	81 (27.7)	Ref.	33 (28.2)	104 (26.6)	Ref.
	Severity									
	Moderate	17 (68.0)	364 (75.4)	Ref.	150 (69.8)	231 (78.8)	Ref.	94 (80.4)	287 (73.4)	Ref.
	Severe	8 (32.0)	119 (24.6)	0.9 (0.4 – 2.2)	65 (30.2)	62 (21.2)	0.6 (0.4 – 0.9)*	23 (19.7)	104 (26.6)	1.4 (0.8 – 2.3)

For peer review only - http://bmjopen.bmj.com/site/about/guidelines.xhtml

Malawi (n=215)	Age group									
	2 – 17	6 (50.0)	40 (19.9)	Ref.	10 (34.5)	35 (19.1)	Ref.	14 (14.0)	33 (29.0)	Ref.
	18-49	5 (41.7)	49 (24.4)	1.5 (0.4 – 5.2)	2 (6.9)	52 (28.4)	7.4 (1.5 – 36.1)*	20 (20.0)	34 (29.8)	0.7 (0.3 – 1.7)
	50+	1 (8.3)	112 (55.7)	18.2 (2.1 – 157.6)*	17 (58.6)	96 (52.5)	1.6 (0.6 – 3.8)	66 (66.0)	47 (41.2)	0.3 (0.1 – 0.6)*
	Gender									
	Male	5 (41.7)	96 (47.8)	Ref.	15 (51.7)	86 (47.0)	Ref.	52 (50.0)	49 (43.0)	Ref.
	Female	7 (58.3)	105 (52.4)	0.6 (0.2 – 2.0)	14 (48.3)	97 (53.0)	1.3 (0.6 – 2.8)	48 (48.0)	65 (57.0)	1.7 (1.0 – 3.1)
	Type									
	Multiple	6 (50.0)	67 (35.1)	Ref.	16 (55.2)	56 (32.4)	Ref.	43 (44.8)	30 (27.8)	Ref.
	Hearing	2 (16.7)	25 (13.1)	1.9 (0.3 – 11.0)	1 (3.5)	26 (15.0)	9.7 (1.1 – 82.1)*	6 (6.3)	21 (19.4)	5.0 (1.7 – 14.8)*
	Seeing	0	42 (22.0)	-	2 (6.9)	40 (23.1)	6.4 (1.4 – 30.4)*	19 (19.8)	23 (21.3)	2.0 (0.9 – 4.6)
	Intellectual	1 (8.3)	13 (6.8)	2.6 (0.3 – 25.4)	3 (10.3)	11 (6.4)	1.5 (0.3 – 7.1)	3 (3.1)	11 (10.2)	4.2 (1.0 – 17.4)
	Physical	3 (25.0)	44 (23.0)	1.1 (0.2 – 5.1)	7 (24.1)	40 (23.1)	1.5 (0.6 – 4.2)	25 (26.0)	23 (21.3)	1.5 (0.7 – 3.2)
	Severity									
	Moderate	7 (58.3)	152 (79.6)	Ref.	14 (48.3)	145 (83.8)	Ref.	62 (64.6)	97 (89.8)	Ref.
	Severe	5 (41.7)	39 (20.4)	0.4 (0.1 – 1.4)	15 (51.7)	28 (16.2)	0.2 (0.1 -0.4)*	34 (35.4)	11 (10.2)	0.2 (0.1 – 0.3)*

*denotes statistical significance at the 95% confidence level

For peer review only - http://bmjopen.bmj.com/site/about/guidelines.xhtml

DISCUSSION

Poorer access to WASH amongst people with disabilities across low and middle income countries is "widely acknowledged but little studied" [15]. This analysis of five cross-sectional, single or multi-district, population-based surveys provides some of the first comparable quantitative data on the relationship between disability and WASH at the individual and household level, including multi-country evidence from two African and two South-Asian settings.

No differences in access to improved latrines were observed between households with and without a person with a disability in any of the five datasets. Households with a person with a disability were more likely to have to spend over thirty minutes (round-trip) fetching water than those without at the Cameroon and Indian sites, and households including a person with a disability were more likely to use a shared sanitation facility in Bangladesh-2. The former finding may suggest that in these two settings households with one or more people with disabilities live further away from centrally located water points (i.e. community peripheries). Secondly, given that the questions were posed directly to people with disabilities in the house, it may reflect their increased time to complete the same journey as others. Shared sanitation facilities of any description were previously considered unimproved by the JMP, due to concerns of privacy and hygiene[30]. However, the 2017 update acknowledges improved shared facilities, and a recent study by Nelson et al. (2014) estimated that 37% of households in Bangladesh (similar to proportions identified in Bangladesh-2 data) utilised a shared but improved facility, and that 75% of households reported satisfaction with the facility[31 32]. This suggests that the use of shared sanitation facilities does not necessarily equate to negative sanitation experience.

Within the household, most people with disabilities used the same sanitation facility as other members of their family, but also frequently reported coming into direct contact with faeces when they did so. Furthermore, the majority of people with disabilities in Bangladesh and almost half in Malawi were unable to collect drinking water from the source themselves. These analyses also showed that older people generally had better access than children, although given the different WASH practices of children and older people it is difficult to know whether or not this was related to disability.

Overall, few differences in WASH access were observed between households that included a member with a disability and those that did not. However, there is a need to better understand the quality of WASH service accessed by people with disabilities, particularly for those whose households do not have access to improved facilities. In India, for example, almost two thirds of people with disabilities interviewed practiced open defecation, and over half of people with disabilities were unable to do this without coming into direct contact with faeces. These findings are of concern as it is well established that direct contact with faeces substantially increases risk of both diarrhoeal disease and hygiene-related stigma [33]. Several recent studies have observed survey respondents' preference for open defecation in rural India even when latrines are available, highlighting the need for clearer and sustained promotion on disability inclusive latrine use in this setting[34 35].

There was evidence that people with disabilities have more difficulties both collecting water themselves and accessing it within their homes across datasets. This has been previously linked to lower volume of water consumption, dehydration and the potential for increased morbidity

For peer review only - http://bmjopen.bmj.com/site/about/guidelines.xhtml

[36]. These findings confirm the need for more nuanced quantitative data collection that captures the quality of access to WASH at the individual level and can inform on appropriate WASH programming. This is important because previous research has suggested that WASH programmes may inadvertently exclude people with disabilities through the delivery of improved, but inaccessible facilities, highlighting the need for better quality monitoring data that records whether facilities are accessible or not [15]. Our findings show that data on WASH and disability should be collected at the individual rather than the household level in order to identify the concerns facing people with disabilities in this domain. Specifically, these findings promote the inclusion of intra-household access questions in the JMP's recommended questions for household surveys, to better understand and overcome lower quality access to WASH amongst persons with disabilities within their households.

Furthermore, additional domains may need to be included in WASH scores, focusing on items such as appropriateness and quality of WASH available with respect to people with disabilities. Ideally, surveys on disability should routinely include individual WASH data as it represents a major challenge facing people with disabilities in low and middle income countries[16].

Several meta-analyses have quantified the implications of improved WASH for exposure pathways for diarrhoeal and infectious diseases, thereby affecting disease burden in "Disability-Adjusted Life Years" (DALYS)[37 38]. However, no previous quantitative studies have explored the implications of disability for access to improved WASH, and in particular the implications for quality of WASH access. A recent qualitative study undertaken in Malawi established that needs and barriers amongst people with disabilities related to WASH varied markedly between individuals, in relation to impairment type, gender and socio-economic factors[16], with much more work needed in this area. Programme implementers and policy makers must ensure that the needs of persons with disabilities are incorporated into programme design in a comprehensive, participatory way that includes intra-household WASH access.

There were a number of limitations to the design of the analyses that need to be taken into account. Whilst response rates across datasets were high, data on the characteristics of non-responders were not available. Disability prevalence estimates varied markedly across the studies, largely because of the different methods and disability definitions used across the surveys. In Cameroon and India, a self-reported measure of disability was combined with clinical screening for specific impairments, and therefore the prevalence was higher there than in the Bangladesh-2 and Malawi studies where the self-reported measure was used in isolation. In the Bangladesh-1 study, a binary screen was applied, which may explain the low prevalence of disability in that survey. Presenting prevalence estimates based on the Washington Group Short Set (WGSS) only, provided similar estimates across settings, although these were higher in the datasets where individuals self-reported (Cameroon and India) compared to those in which a household head reported on behalf of the rest of the household (Bangladesh-2 and Malawi). This finding supports the ongoing dialogue regarding the importance of comparable disability measures for use in population-based surveys, including in the measurement of childhood disability, the international recommendation for which is the use of tools by the Washington Group on Disability Statistics [39 40]. Furthermore, each survey was conducted at the state or regional level and the estimates derived therefore do not constitute national estimates. Finally, comparative data on indicators of quality access to WASH amongst controls/ people without disabilities were not collected in any of the studies. There were also important strengths. The

For peer review only - http://bmjopen.bmj.com/site/about/guidelines.xhtml

surveys were relatively large, conducted in different geographic areas, and collected broadly consistent information on WASH.

CONCLUSION

No relationship was observed between household-level WASH access and the presence of a household member with a disability in five surveys conducted in low and middle income countries. However, at the individual level, whilst most people with disabilities could access the same facilities as other members of their household, this often entailed contact with faeces and required assistance from others. In particular, people who reported severe limitations experienced the least equitable intra-household WASH access. Type of limitation reported, age and gender were also predictive factors for inequitable access in certain cases. Consequently, the quality of the WASH access is likely to have been poorer among people with disabilities. This requires programmatic and research attention. Future data collection should capture quality of WASH access in a more nuanced way and collect individual level data from people with and without disabilities to make accurate comparisons. Further programmatic work is needed to improve general access to WASH, and this must be inclusive in nature to ensure that the quality of access is equitable among people with disabilities.

ACKNOWLEDGEMENTS

We gratefully acknowledge the contribution of our colleague the late James Milner. His untimely and tragic death during fieldwork in Malawi was a great loss to his friends and colleagues as well as to the scientific research community in Malawi and beyond.

FUNDING

This research has been funded in part by the Australian Aid (Australian Government Department of Foreign Affairs and Trade) through the Australian Development Research Awards Scheme under an award entitled 'Disability and its impact on safe hygiene and sanitation. The views expressed in this publication are those of the authors and not necessarily those of the Commonwealth of Australia. The Commonwealth of Australia accepts no responsibility for any loss, damage or injury resulting from reliance on any of the information or views contained in this publication.

Bangladesh-1: Funded by The Bill & Melinda Gates Foundation, Global Development Grant Number OPP1102989.

Bangladesh-2: Funded by Australian Aid (Australian Government Department of Foreign Affairs and Trade), Grant number 66469

India and Cameroon: Funded by CBM Germany, LSHTM Grant Number ITCRRH71

Malawi: Funded by Australian Aid (Australian Government Department of Foreign Affairs and Trade), Grant number 66469

COMPETING INTERESTS

There were no competing interests.

For peer review only

AUTHOR'S CONTRIBUTIONS

All authors reviewed the final draft of the manuscript and agree with the findings and conclusions.

IM was responsible for drafting the paper, conducting the data analysis, and overseeing the fieldwork in India and Cameroon.

WPS gave oversight on the data analysis and feedback on all manuscript drafts

KB contributed to the study design of the Bangladesh-2 dataset and reviewed manuscript drafts

JC oversaw data collection for the Malawi dataset

LD contributed to the study design of the Malawi dataset, oversaw data collection for the Malawi dataset and reviewed manuscript drafts

AKH was responsible for the study design of the Bangladesh-1 dataset, oversaw data collection and data management for the Bangldesh-1 dataset and reviewed manuscript drafts

SPJ contributed to the study design of the Bangladesh-2 dataset, oversaw data collection for the Bangladesh-2 dataset and reviewed manuscript drafts

SP was responsible for the study design and data collection on India and Cameroon.

MR contributed to the study design of the Bangladesh-2 dataset

MS contributed to the study design of the Bangladesh-2 dataset

HK assisted in drafting the paper, and guided the data analysis, and contributed towards designing the study in India and Cameroon.

AB was responsible for the study design of the Malawi dataset, contributed to the design of the Bangladesh-2 dataset, gave oversight on data analysis and feedback on all manuscript drafts, and coordinated access to external datasets. AB also contributed to the design of the data collection tool across all study sites.

DATA SHARING STATEMENT

Data for each dataset is available from the respective project leads on request.

REFERENCES

1. Johnston EA, Teague J, Graham JP. Challenges and opportunities associated with neglected tropical disease and water, sanitation and hygiene intersectoral integration programs. *BMC Public Health* 2015;15(1):547.
2. Prüss-Ustün A, Bartram J, Clasen T, et al. Burden of disease from inadequate water, sanitation and hygiene in low-and middle-income settings: a retrospective analysis of data from 145 countries. *Tropical Medicine & International Health* 2014;19(8):894-905.
3. Freeman MC, Ogden S, Jacobson J, et al. Integration of water, sanitation, and hygiene for the prevention and control of neglected tropical diseases: a rationale for inter-sectoral collaboration. *PLoS Negl Trop Dis* 2013;7(9):e2439.

For peer review only - http://bmjopen.bmj.com/site/about/guidelines.xhtml

4. Mara DD. Water, sanitation and hygiene for the health of developing nations. *Public health* 2003;117(6):452-56.

5. United Nations. Transforming our world: the 2030 Agenda for Sustainable Development. Geneva: United Nations General Assembly, 2015.

6. UNICEF and the World Health Organisation. 25 years Progress on Sanitation and Drinking Water: 2015 Update and MDG Assessment. United States of America: Available from: http://www.wssinfo.org/fileadmin/user_upload/resources/JMP-Update-report-2015_English.pdf, 2015.

7. WHO / UNICEF JMP. Refining the definitions: an ongoing process and the ladder concept Available from: http://www.wssinfo.org/definitions-methods/2015 [2015.

8. Lim SS, Vos T, Flaxman AD, et al. A comparative risk assessment of burden of disease and injury attributable to 67 risk factors and risk factor clusters in 21 regions, 1990–2010: a systematic analysis for the Global Burden of Disease Study 2010. *The lancet* 2013;380(9859):2224-60.

9. Schmidt WP. The elusive effect of water and sanitation on the global burden of disease. *Tropical Medicine & International Health* 2014;19(5):522-27.

10. Bullinger M, Schmidt S, Petersen C, et al. Assessing quality of life of children with chronic health conditions and disabilities: a European approach. *International Journal of Rehabilitation Research* 2002;25(3):197-206.

11. United Nations. Convention of the Rights of Persons with Disabilities and Optional Protocol. New York: United Nations, 2006.

12. World Health Organization. World Report on Disability Geneva: World Health Organization 2011.

13. Mitra S, Pošarac A, Vick B. Disability and poverty in developing countries: a snapshot from the world health survey: World Bank 2011.

14. Mactaggart I, Kuper H, Murthy G, et al. Assessing health and rehabilitation needs of people with disabilities in Cameroon and India. *Disability and rehabilitation* 2015:1-8.

15. Groce N, Bailey N, Lang R, et al. Water and sanitation issues for persons with disabilities in low- and middle-income countries: a literature review and discussion of implications for global health and international development. *Journal of water and health* 2011;9(4):617-27.

16. White S, Kuper H, Itimu-Phiri A, et al. A Qualitative Study of Barriers to Accessing Water, Sanitation and Hygiene for Disabled People in Malawi. *PloS one* 2016;11(5):e0155043.

17. Moe CL, Rheingans RD. Global challenges in water, sanitation and health. *Journal of water and health* 2006;4(S1):41-57.

18. Mactaggart I, Schmidt WP, Bostoen K, et al. Supplementary File 1: Ethical Review Bodies. Access to water and sanitation amongst people with disabilities: results from cross-sectional surveys in Bangladesh, Cameroon, India and Malawi *Plos One* 2018

19. Washington Group on Disability Statistics. Understanding and interpreting disability as measured using the WG short set of questions. Hyattsville, 2009.

20. Secretariat to the Convention on the Rights of Persons with Disabilities. Final Report on United Nations Expert Group Meeting on Disability Data and Statistics, Monitoring and Evaluation: The Way Forward - a Disability-Inclusive Agenda Towards 2015 and Beyond; Paris July 8-10 2014. Available from http://www.un.org/disabilities/documents/egm2014/EGM_FINAL_08102014.pdf [first accessed 04.01.15], 2014.

21. Atijosan O, Kuper H, Rischewski D, et al. Musculoskeletal impairment survey in Rwanda: design of survey tool, survey methodology, and results of the pilot study (a cross sectional survey). *BMC musculoskeletal disorders* 2007;8(1):30.

22. Lindfield R, Griffiths U, Bozzani F, et al. A rapid assessment of avoidable blindness in Southern Zambia United States: International Centre for Eye Health, London School of Hygiene and Tropical Medicine-LSHTM, London, United Kingdom. Robert.lindfield@lshtm.ac.uk; 2012 [6:[e38483]. Available from:

For peer review only

For peer review only - http://bmjopen.bmj.com/site/about/guidelines.xhtml

http://ovidsp.ovid.com/ovidweb.cgi?T=JS&PAGE=reference&D=medl&NEWS=N&AN=22737211 accessed 101285081 7.
23. Smith A, Mackenzie I. WHO Ear and Hearing Disorders Survey Protocol. Geneva: World Health Organization, 1999.
24. Kroenke K, Spitzer RL. The PHQ-9: a new depression diagnostic and severity measure. *Psychiatr Ann* 2002;32(9):1-7.
25. UNICEF and the World Health Organisation. Core questions on drinking-water and sanitation for household surveys. Switzerland, available from: http://www.wssinfo.org/fileadmin/user_upload/resources/1268174016-JMP_Core_Questions.pdf, 2006.
26. Meg Satterthwaite. JMP Working Group on Equity and Non-Discrimination Final Report. JMP Working Group on Equity and Non-Discrimination (END), available from: http://www.wssinfo.org/fileadmin/user_upload/resources/JMP-END-WG-Final-Report-20120821.pdf, 2012.
27. StataCorp L. Stata 12.0. *Statistics/Data Analysis* 2011;4905
28. Vyas S, Kumaranayake L. Constructing socio-economic status indices: how to use principal components analysis. *Health policy and planning* 2006;21(6):459-68.
29. BRAC. Water, Sanitation and Hygiene: Nine years of scale and innovation in Bangladesh Programme Report 2006-2015. Available from http://www.brac.net/images/WASH/BRACWASHProgrammeFinalReport20062015.pdf [accessed 18.09.17], 2016.
30. Heijnen M, Cumming O, Peletz R, et al. Shared sanitation versus individual household latrines: a systematic review of health outcomes. *PLoS One* 2014;9(4):e93300.
31. Nelson KB, Karver J, Kullman C, et al. User perceptions of shared sanitation among rural households in Indonesia and Bangladesh. *PloS one* 2014;9(8):e103886.
32. Organization WH, UNICEF. Progress on drinking water, sanitation and hygiene: 2017 update and SDG baselines. 2017
33. Murray CJ, Lopez AD. Global mortality, disability, and the contribution of risk factors: Global Burden of Disease Study. *The Lancet* 1997;349(9063):1436-42.
34. Coffey D, Gupta A, Hathi P, et al. Revealed preference for open defecation. *Economic & Political Weekly* 2014;49(38):43.
35. Gertler P, Shah M, Alzua ML, et al. How does health promotion work? Evidence from the dirty business of eliminating open defecation: National Bureau of Economic Research, 2015.
36. Manz F, Wentz A. The importance of good hydration for the prevention of chronic diseases. *Nutrition reviews* 2005;63(6):S2.
37. Prüss A, Kay D, Fewtrell L, et al. Estimating the burden of disease from water, sanitation, and hygiene at a global level. *Environmental health perspectives* 2002;110(5):537-42.
38. Gleick PH. Dirty-water: Estimated Deaths from Water-related Diseases 2000-2020: Citeseer 2002.
39. United Nations. Improvement of disability data and statistics: objectives and challenges. Conference of State Parties to the Convention on the Rights of Persons with Disabilities Eighth Session, New York 9-11 June 2015, 2015.
40. Palmer MH, D. Models and Measurement in disability: an international review. *Health Policy and Planning* 2011:1-8.

For peer review only - http://bmjopen.bmj.com/site/about/guidelines.xhtml

Supplementary File 1: Ethical Approval obtained for each study

Bangladesh-1:

- Emory University Internal Review Board (Georgia, USA)
- Icddr,b Internal Review Board (Dhaka, Bangladesh)

Bangladesh-2:

- James P Grant School of Public Health Ethical Review Committee, BRAC University (Dhaka, Bangladesh)

Cameroon:

- The London School of Hygiene and Tropical Medicine Observational/ Interventions Research Ethics Committee (London, UK)
- National Ethics Committee for Research in Human Health (CNERSH, Cameroon)
- Cameroon Baptist Convention Health Board Institutional Review Board (Cameroon)

India:

- The London School of Hygiene and Tropical Medicine Observational/ Interventions Research Ethics Committee (London, UK)
- Indian Institute of Public Health Hyderabad Institutional Ethics Committee (India)
- Government of India Health Ministry Screening Committee (India)

Malawi:

- The London School of Hygiene and Tropical Medicine Observational/ Interventions Research Ethics Committee (London, UK)
- National Commission for Science and Technology (Lilongwe, Malawi)

For peer review only

Appendix 1: Formulation of the JMP Questions and disability prevalence questions used in each data set.

Bangladesh 1	
Household head report	
Question	Responses
Does this person have a disability?	Yes<br>No
If yes, type of disability	Disability caused by Polio<br>Clubfoot<br>Effects of Leprosy<br>Brain Injury and Cerebral Palsy<br>Scoliosis (hunch back)<br>Spinal Cord Injury<br>Birth defects<br>Dwarfism and Gigantism<br>Injury, burns and amputations<br>Spinal Bifida (swelling of the spinal cord)<br>Muscular Dystrophy and Muscular Atrophy (progressive weakness of muscles and body function)<br>Juvenile Arthritis, Rheumatism and other joint pains<br>Bone infections (including tuberculosis of the spine<br>Hip problems<br>Elephantiasis.<br>Mental disability<br>Disable due to overaged<br>Disable due to blind<br>Disable due to deaf<br>Disable due to paralysis<br>Others including multiple disabilities (specify)
Does the household have any access to any latrines?	Yes

For peer review only

	No
If yes, type of toilet facility	**(Improved sanitation facilities)**<br>[Flush or pour flush to]:<br>Flush to pit<br>Flush to Septic tank<br>Piped sewer system/ Flush to elsewhere<br>Pit latrine with slab & water seal<br>Ventilated Improved Pit (VIP) latrine<br>Composting toilet<br><br>**(Unimproved sanitation facilities)**<br>Open pit latrine<br>Hanging toilet/latrine<br>Others: Specify
Ownership of toilet facility	Household owns toilet<br>Household Shares Ownership of Toilet<br>Public<br>Other
Person with a disability self report	
Do you use the same toilet facility as other members of your household	Yes<br>No
If no, why do you use a different toilet facility from other members of your household	It is physically impossible or difficult for me to use the same facility<br>I am not allowed to use the same facility/ other people would not like it<br>I might face physical or verbal abuse<br>I would be embarrassed<br>Other [specify]
Are you usually able to use the toilet facility without you or your clothing coming into contact with faeces	Yes<br>No
Do you collect water for drinking by yourself	Yes<br>No

For peer review only

Do you able collect drinking water without assistance from another person	Yes<br><br>No
Do you use the same facility / place for bathing as other members of your household	Yes<br><br>No

Bangladesh 2	
Household head report	
Question	Responses
Does (x) have difficulty seeing, even if wearing glasses?	No - no difficulty<br>Yes – some difficulty<br>Yes – a lot of difficulty<br>Cannot do at all
Does (x) have difficulty walking or climbing steps?	No - no difficulty<br>Yes – some difficulty<br>Yes – a lot of difficulty<br>Cannot do at all
Does (x) have difficulty (with self-care such as) washing all over or dressing?	No - no difficulty<br>Yes – some difficulty<br>Yes – a lot of difficulty<br>Cannot do at all
Does (x) have difficulty remembering or concentrating?	No - no difficulty<br>Yes – some difficulty<br>Yes – a lot of difficulty<br>Cannot do at all
Does (x) have difficulty hearing, even if using a hearing aid?	No - no difficulty<br>Yes – some difficulty<br>Yes – a lot of difficulty<br>Cannot do at all
Using their usual (customary) language, does (x) have difficulty communicating, for example: understanding or being understood?	No - no difficulty<br>Yes – some difficulty<br>Yes – a lot of difficulty<br>Cannot do at all
What is the main source of drinking water for members of your households?	Piped water into dwelling<br>Piped water into yard/plot<br>Public tap/standpipe<br>Tube well<br>Bore hole<br>Protected well<br>Unprotected well<br>Protected spring<br>Unprotected spring<br>Rainwater<br>Tanker truck<br>Cart with small tank<br>Surface water<br>Bottled water

For peer review only

	Other: specify
How many minute does it take to go to the collection point, get water, and come back? (might be 0 min if piped water into dwelling, plot or yard)	No of minutes
What kind of toilet facility do members of your household usually use?	Flush or pour flush to piped sewer /septic tank/pit latrine<br>Flush or pour flush to elsewhere/unknown<br>Ventilated improved pit latrine<br>Pit latrine with slab<br>Pit latrine without slab/open pit<br>Composting toilet<br>Bucket<br>Hanging toilet/ hanging latrine<br>No facilities or bush or field<br>Other specify
Do you share this facility with other households?	No<br>Yes, with neighbours and other people I know (Shared toilet)<br>Yes, with people I do not know (Public toilet)<br>I do not know
Person with a disability self report	
Where do you go for defecation?	Same toilet as other members go<br>Neighbours or public latrine<br>Open place<br>In the room by using a bucket/ bedpan/potty<br>Other: specify_______________________

Cameroon and India	
Age 2-7 – Caregiver Reported; age 8-17 self reported	
Does [name] have difficulty seeing, even when wearing his/her glasses?	No - no difficulty<br>Yes – some difficulty<br>Yes – a lot of difficulty<br>Cannot do at all
Does [name] have difficulty hearing, even when using his/her hearing aid(s)?	No - no difficulty<br>Yes – some difficulty<br>Yes – a lot of difficulty<br>Cannot do at all
Compared with children of the same age, does [name] have difficulty walking	No - no difficulty<br>Yes – some difficulty<br>Yes – a lot of difficulty<br>Cannot do at all
Age 2-4 Does [name] have difficulty understanding you?	No - no difficulty<br>Yes – some difficulty<br>Yes – a lot of difficulty

For peer review only

	Cannot do at all
Age 2-4 Do you have difficulty understanding what your child wants?	No - no difficulty<br>Yes – some difficulty<br>Yes – a lot of difficulty<br>Cannot do at all
Age 5+ Compared with children of the same age and using [his/her] usual language, does [name] have difficulty understanding other people	No - no difficulty<br>Yes – some difficulty<br>Yes – a lot of difficulty<br>Cannot do at all
Age 5+ Compared with children of the same age and using [his/her] usual language,<br>does [name] have difficulty being understood by other people?	No - no difficulty<br>Yes – some difficulty<br>Yes – a lot of difficulty<br>Cannot do at all
Age 5+ Compared with children of the same age, does [name] have difficulty with self-care<br>such as feeding or dressing him/herself?	No - no difficulty<br>Yes – some difficulty<br>Yes – a lot of difficulty<br>Cannot do at all
Age 2-3 Compared with children of the same age, does [name] have difficulty learning<br>the names of common objects?	No - no difficulty<br>Yes – some difficulty<br>Yes – a lot of difficulty<br>Cannot do at all
Age 3+ Compared with children of the same age, does [name] have difficulty learning to do new things?	No - no difficulty<br>Yes – some difficulty<br>Yes – a lot of difficulty<br>Cannot do at all
Age 5+ Compared with children of the same age, does [name] have difficulty remembering things that they have learned?	No - no difficulty<br>Yes – some difficulty<br>Yes – a lot of difficulty<br>Cannot do at all
Age 18+ Individual Self Report	
Question	Responses
Do you have difficulty seeing, even if wearing glasses?	No - no difficulty<br>Yes – some difficulty<br>Yes – a lot of difficulty<br>Cannot do at all
Do you have difficulty hearing, even if using a hearing aid?	No - no difficulty<br>Yes – some difficulty<br>Yes – a lot of difficulty<br>Cannot do at all
Do you have difficulty walking or climbing steps?	No - no difficulty<br>Yes – some difficulty<br>Yes – a lot of difficulty<br>Cannot do at all
Do you have difficulty remembering or concentrating?	No - no difficulty<br>Yes – some difficulty<br>Yes – a lot of difficulty

For peer review only

	Cannot do at all
Do you have difficulty (with self-care such as) washing al over or dressing?	No - no difficulty<br>Yes – some difficulty<br>Yes – a lot of difficulty<br>Cannot do at all
Using their usual (customary) language, does (x) have difficulty communicating, for example: understanding or being understood?	No - no difficulty<br>Yes – some difficulty<br>Yes – a lot of difficulty<br>Cannot do at all
Do you have difficulty raising a 2 liter bottle of water or soda from waist to eye level?	No - no difficulty<br>Yes – some difficulty<br>Yes – a lot of difficulty<br>Cannot do at all
Do you have difficulty using [your/his/her] hands and fingers, such as picking up small objects, for example, a button or pencil, or opening or closing containers or bottles?	No - no difficulty<br>Yes – some difficulty<br>Yes – a lot of difficulty<br>Cannot do at all
What is the main source of drinking water for members of your households?	Piped water into dwelling<br>Piped water into yard/plot<br>Public tap/standpipe<br>Tube well<br>Bore hole<br>Protected well<br>Unprotected well<br>Protected spring<br>Unprotected spring<br>Rainwater<br>Tanker truck<br>Cart with small tank<br>Surface water<br>Bottled water<br>Other: specify
Vision Screen	Presenting Visual Acuity <6/18 in better eye
Hearing Screen	Average Pure Tone Audiometry A-weighted decibel reading of >35 in better ear (children) or >40 (adults)
Musculoskeletal impairment screen	Physiotherapist observed effects on the ability of the musculoskeletal system to function as a whole, categorized as moderate or severe
Clinical Depression (18+)	Composite score of 20+ using Patient Health Questionnaire (PHQ9)
What kind of toilet facility do members of your household usually use?	Flush toilet<br>Traditional latrine<br>Ventilation improved pit latrine<br>Bowl/Bucket<br>No Toilet<br>Other: specify
Do you share this facility with other households?	Used only by your household

For peer review only

	Shared with other households<br>Public /Communal/ Community latrine
Do you use the same toilet facility as other members of your household	Yes<br>No
If no, why not?	It would be physically impossible<br>I'm not allowed/others would not like it<br>I might face verbal or physical abuse<br>I would be embarrassed<br>Other: specify
Are you usually able to use the toilet facility without you or your clothing coming into contact with faeces	Yes<br><br>No
What is the main source of drinking water for members of your households?	Private Pipeline<br>Private well<br>Public tap/standpipe<br>Public well<br>Neighbours<br>Water Vendor<br>Spring<br>River/stream/lake<br>Rainwater<br>Other: Specify
How long does it take to go there, get water and come back?	Number of minutes
Do you collect water for drinking?	Yes<br>No
Are you able to access drinking water at home without assistance?	Yes<br>No
Have you had diarrhea in the past four weeks?	Yes<br>No

Malawi	
Age 2-7 – Caregiver Reported; age 8-17 self reported	
Does [name] have difficulty seeing, even when wearing his/her glasses?	No - no difficulty<br>Yes – some difficulty<br>Yes – a lot of difficulty<br>Cannot do at all
Does [name] have difficulty hearing, even when using his/her hearing aid(s)?	No - no difficulty<br>Yes – some difficulty<br>Yes – a lot of difficulty<br>Cannot do at all
Compared with children of the same age, does [name] have difficulty walking	No - no difficulty<br>Yes – some difficulty<br>Yes – a lot of difficulty

For peer review only

	Cannot do at all
Age 2-4 Does [name] have difficulty understanding you?	No - no difficulty<br>Yes – some difficulty<br>Yes – a lot of difficulty<br>Cannot do at all
Age 2-4 Do you have difficulty understanding what your child wants?	No - no difficulty<br>Yes – some difficulty<br>Yes – a lot of difficulty<br>Cannot do at all
Age 5+ Compared with children of the same age and using [his/her] usual language, does [name] have difficulty understanding other people	No - no difficulty<br>Yes – some difficulty<br>Yes – a lot of difficulty<br>Cannot do at all
Age 5+ Compared with children of the same age and using [his/her] usual language,<br>does [name] have difficulty being understood by other people?	No - no difficulty<br>Yes – some difficulty<br>Yes – a lot of difficulty<br>Cannot do at all
Age 5+ Compared with children of the same age, does [name] have difficulty with self-care<br>such as feeding or dressing him/herself?	No - no difficulty<br>Yes – some difficulty<br>Yes – a lot of difficulty<br>Cannot do at all
Age 2-3 Compared with children of the same age, does [name] have difficulty learning<br>the names of common objects?	No - no difficulty<br>Yes – some difficulty<br>Yes – a lot of difficulty<br>Cannot do at all
Age 3+ Compared with children of the same age, does [name] have difficulty learning to do new things?	No - no difficulty<br>Yes – some difficulty<br>Yes – a lot of difficulty<br>Cannot do at all
Age 5+ Compared with children of the same age, does [name] have difficulty remembering things that they have learned?	No - no difficulty<br>Yes – some difficulty<br>Yes – a lot of difficulty<br>Cannot do at all
Age 18+ Individual Self Report if present or household head report if not	
Question	Responses
Do you have difficulty seeing, even if wearing glasses?	No - no difficulty<br>Yes – some difficulty<br>Yes – a lot of difficulty<br>Cannot do at all
Do you have difficulty hearing, even if using a hearing aid?	No - no difficulty<br>Yes – some difficulty<br>Yes – a lot of difficulty<br>Cannot do at all
Do you have difficulty walking or climbing steps?	No - no difficulty<br>Yes – some difficulty<br>Yes – a lot of difficulty

For peer review only

	Cannot do at all
Do you have difficulty remembering or concentrating?	No - no difficulty<br>Yes – some difficulty<br>Yes – a lot of difficulty<br>Cannot do at all
Do you have difficulty (with self-care such as) washing al over or dressing?	No - no difficulty<br>Yes – some difficulty<br>Yes – a lot of difficulty<br>Cannot do at all
Using their usual (customary) language, does (x) have difficulty communicating, for example: understanding or being understood?	No - no difficulty<br>Yes – some difficulty<br>Yes – a lot of difficulty<br>Cannot do at all
Do you have difficulty raising a 2 liter bottle of water or soda from waist to eye level?	No - no difficulty<br>Yes – some difficulty<br>Yes – a lot of difficulty<br>Cannot do at all
Do you have difficulty using [your/his/her] hands and fingers, such as picking up small objects, for example, a button or pencil, or opening or closing containers or bottles?	No - no difficulty<br>Yes – some difficulty<br>Yes – a lot of difficulty<br>Cannot do at all
Does your household have a latrine?	Yes<br>No
If yes, what type of latrine is it?	Flush to pit<br>Ventilation improved pit latrine<br>Pit latrine without slab<br>Pit latrine without slab/Open pit<br>Other: specify
Do you share this facility with other households?	Used only by your household<br>Shared with other households<br>Public /Communal/ Community latrine
Do you use the same toilet facility as other members of your household	Yes<br>No
If no, why not?	It would be physically impossible<br>I'm not allowed/others would not like it<br>I might face verbal or physical abuse<br>I would be embarrassed<br>Other: specify
Are you usually able to use the toilet facility without you or your clothing coming into contact with faeces or urine	Yes<br><br>No
What is the main source of drinking water for members of your household?	Piped into dwelling<br>Piped into compound, yard or plot<br>Piped into neighbours<br>Public tap/standpipe<br>Tubewell/borewell<br>Protected well<br>Unprotected well<br>Protected Spring

For peer review only

	Unprotected Spring<br>Rainwater<br>Tanker Truck<br>Cart with small tank/drum<br>Surface Water<br>Bottled Water<br>Other: Specify
How long does it take for someone to walk to the water source, collect water and come back?	Inside house<br><15 minutes<br>15 – 30 minutes<br>30 minutes – 1 hour<br>>1 hour
Do you collect water for drinking, cooking and other purposes such as for bathing, hand washing and cleaning?	Yes<br>No
If no, why not?	It would be physically impossible<br>I'm not allowed/others would not like it<br>I might face verbal or physical abuse<br>I would be embarrassed<br>Other: specify
Are you able to access drinking water at home without assistance?	Yes<br>No
Has anyone in your household had diarrhea in the past 7 days?	Yes<br>No

For peer review only

STROBE Statement—checklist of items that should be included in reports of observational studies

	Item No	Recommendation
**Title and abstract**	1	(*a*) Indicate the study's design with a commonly used term in the title or the abstract **[within Title page and Design section of abstract]**
		(*b*) Provide in the abstract an informative and balanced summary of what was done and what was found **[see results section of abstract p2]**
**Introduction**		
Background/rationale	2	Explain the scientific background and rationale for the investigation being reported **[page 4]**
Objectives	3	State specific objectives, including any prespecified hypotheses **[page 4]**
**Methods**		
Study design	4	Present key elements of study design early in the paper **[page 5]**
Setting	5	Describe the setting, locations, and relevant dates, including periods of recruitment, exposure, follow-up, and data collection **[page 5-7]**
Participants	6	(*a*) *Cohort study*—Give the eligibility criteria, and the sources and methods of selection of participants. Describe methods of follow-up **[N/A]**<br>*Case-control study*—Give the eligibility criteria, and the sources and methods of case ascertainment and control selection. Give the rationale for the choice of cases and controls **[page 5-7]**<br>*Cross-sectional study*—Give the eligibility criteria, and the sources and methods of selection of participants **[page 5-7]**
		(*b*) *Cohort study*—For matched studies, give matching criteria and number of exposed and unexposed **[N/A]**<br>*Case-control study*—For matched studies, give matching criteria and the number of controls per case **[page 5-7]**
Variables	7	Clearly define all outcomes, exposures, predictors, potential confounders, and effect modifiers. Give diagnostic criteria, if applicable **[page 5-7]**
Data sources/ measurement	8*	For each variable of interest, give sources of data and details of methods of assessment (measurement). Describe comparability of assessment methods if there is more than one group **[page 5-7]**
Bias	9	Describe any efforts to address potential sources of bias **[page 7]**
Study size	10	Explain how the study size was arrived at **[page 7]**
Quantitative variables	11	Explain how quantitative variables were handled in the analyses. If applicable, describe which groupings were chosen and why **[page 8]**
Statistical methods	12	(*a*) Describe all statistical methods, including those used to control for confounding **[page 8]**
		(*b*) Describe any methods used to examine subgroups and interactions **[page 8]**
		(*c*) Explain how missing data were addressed **[page 8]**
		(*d*) *Cohort study*—If applicable, explain how loss to follow-up was addressed **[N/A]**<br>*Case-control study*—If applicable, explain how matching of cases and controls was addressed **[page 5-7]**<br>*Cross-sectional study*—If applicable, describe analytical methods taking account of sampling strategy**[page 5-7]**
		(*e*) Describe any sensitivity analyses **[N/A]**

Continued on next page

For peer review only - http://bmjopen.bmj.com/site/about/guidelines.xhtml

**Results**		
Participants	13*	(a) Report numbers of individuals at each stage of study—eg numbers potentially eligible, examined for eligibility, confirmed eligible, included in the study, completing follow-up, and analysed **[page 7, page 9]**
		(b) Give reasons for non-participation at each stage **[page 9]**
		(c) Consider use of a flow diagram **[N/A information in table 2]**
Descriptive data	14*	(a) Give characteristics of study participants (eg demographic, clinical, social) and information on exposures and potential confounders **[Table 2, page 9]**
		(b) Indicate number of participants with missing data for each variable of interest **[provided in relevant tables]**
		(c) *Cohort study*—Summarise follow-up time (eg, average and total amount) **[N/A]**
Outcome data	15*	*Cohort study*—Report numbers of outcome events or summary measures over time **[N/A]**
		Case-control study—Report numbers in each exposure category, or summary measures of exposure **[Table 2, page 9]**
		Cross-sectional study—Report numbers of outcome events or summary measures **[Table 2, 3, 4,5, 6 page 9-15]**
Main results	16	(*a*) Give unadjusted estimates and, if applicable, confounder-adjusted estimates and their precision (eg, 95% confidence interval). Make clear which confounders were adjusted for and why they were included **[Table 2, 3, 4,5,6 page 9-15]**
		(*b*) Report category boundaries when continuous variables were categorized **[Table 2, 3, 4,5,6 page 9-15]**
		(*c*) If relevant, consider translating estimates of relative risk into absolute risk for a meaningful time period **[N/A]**
Other analyses	17	Report other analyses done—eg analyses of subgroups and interactions, and sensitivity analyses **[N/A]**
**Discussion**		
Key results	18	Summarise key results with reference to study objectives **[page 16-17]**
Limitations	19	Discuss limitations of the study, taking into account sources of potential bias or imprecision. Discuss both direction and magnitude of any potential bias **[page 17]**
Interpretation	20	Give a cautious overall interpretation of results considering objectives, limitations, multiplicity of analyses, results from similar studies, and other relevant evidence **[page 17]**
Generalisability	21	Discuss the generalisability (external validity) of the study results **[page 17]**
**Other information**		
Funding	22	Give the source of funding and the role of the funders for the present study and, if applicable, for the original study on which the present article is based **[page 17]**

*Give information separately for cases and controls in case-control studies and, if applicable, for exposed and unexposed groups in cohort and cross-sectional studies.

Note: An Explanation and Elaboration article discusses each checklist item and gives methodological background and published examples of transparent reporting. The STROBE checklist is best used in conjunction with this article (freely available on the Web sites of PLoS Medicine at http://www.plosmedicine.org/, Annals of Internal Medicine at http://www.annals.org/, and Epidemiology at http://www.epidem.com/). Information on the STROBE Initiative is available at www.strobe-statement.org.

For peer review only
